# Virus–Host Protein Interaction Network of the Hepatitis E Virus ORF2-4 by Mammalian Two-Hybrid Assays

**DOI:** 10.3390/v15122412

**Published:** 2023-12-12

**Authors:** Laura Corneillie, Irma Lemmens, Karin Weening, Amse De Meyer, Freya Van Houtte, Jan Tavernier, Philip Meuleman

**Affiliations:** 1 Laboratory of Liver Infectious Diseases, Department of Diagnostic Sciences, Faculty of Medicine and Health Sciences, Ghent University, 9000 Ghent, Belgium; 2 VIB-UGent Center for Medical Biotechnology, Department of Biomolecular Medicine, Faculty of Medicine and Health Sciences, Ghent University, 9000 Ghent, Belgium

**Keywords:** protein–protein interactions, MAPPIT, KISS, viral hepatitis, host factor, hepatitis E virus, virus–host interaction, SHARPIN, RNF5, antiviral signaling

## Abstract

Throughout their life cycle, viruses interact with cellular host factors, thereby influencing propagation, host range, cell tropism and pathogenesis. The hepatitis E virus (HEV) is an underestimated RNA virus in which knowledge of the virus–host interaction network to date is limited. Here, two related high-throughput mammalian two-hybrid approaches (MAPPIT and KISS) were used to screen for HEV-interacting host proteins. Promising hits were examined on protein function, involved pathway(s), and their relation to other viruses. We identified 37 ORF2 hits, 187 for ORF3 and 91 for ORF4. Several hits had functions in the life cycle of distinct viruses. We focused on SHARPIN and RNF5 as candidate hits for ORF3, as they are involved in the RLR-MAVS pathway and interferon (IFN) induction during viral infections. Knocking out (KO) SHARPIN and RNF5 resulted in a different IFN response upon ORF3 transfection, compared to wild-type cells. Moreover, infection was increased in SHARPIN KO cells and decreased in RNF5 KO cells. In conclusion, MAPPIT and KISS are valuable tools to study virus–host interactions, providing insights into the poorly understood HEV life cycle. We further provide evidence for two identified hits as new host factors in the HEV life cycle.

## 1. Introduction

During its life cycle, viruses interact with a variety of host proteins for their survival, pathogenesis and virus spread. Unraveling virus–host interactions at the protein level aids in the understanding of a virus’ life cycle, its molecular virology and immunology. Moreover, insight into these complex interactions will indicate potential antiviral targets and therefore, protein–protein interactions (PPIs) offer a wide range of possibilities to develop new and more effective therapies.

Many technologies to discover PPIs have been developed, ranging from two-hybrid systems to mass spectrometry, phage display and protein chip technology; all function in high-throughput formats [1,2]. Two described analogous high-throughput mammalian two-hybrid assays are the MAmmalian Protein–protein Interaction Trap (MAPPIT) and the KInase Substrate Sensor (KISS) [2,3]. MAPPIT and KISS are complementation assays based on (parts) of the JAK-STAT signaling pathway. In MAPPIT, a bait protein of interest is fused to a type I transmembrane cytokine receptor that is mutated at its STAT recruitment sites. A prey protein is fused to the cytoplasmic portion of another cytokine receptor containing intact STAT docking sites. Upon the functional association of bait and prey, the JAK proteins can phosphorylate the prey-linked moiety in trans, leading to subsequent STAT binding, dimerization and translocation to the nucleus to induce reporter gene activation. In KISS, a bait protein of interest is fused to the tyrosine kinase 2 (TYK2) domain, which after interaction with the prey is able to phosphorylate the prey-linked moiety. This results in the same downstream signaling as with MAPPIT, but it overcomes the tethering of the bait moiety to the plasma membrane. The advantage of those mammalian systems is that they allow for temporal, spatial and functional modulation and that necessary co-factors or regulatory proteins to study those dynamics of PPIs are present. Moreover, proteins can undergo the proper modifications in their native cellular environment, which is often necessary to allow interactions. The sensitivity and specificity of the described methods are comparable to that of other well-known assays such as Yeast-Two-Hybrid (Y2H) [4]. Both MAPPIT and KISS have shown in the past to be robust methods for detecting PPIs for a variety of applications, including in the field of virus research [5,6].

In this study, both systems were used to identify novel protein binding partners for the proteins of the hepatitis E virus (HEV). HEV is a positive-sense, single-stranded RNA virus and currently the major cause of acute hepatitis worldwide, affecting about 20 million people each year [7]. No specific therapy is available today and a vaccine is only licensed in China [8], highlighting the need for new treatment options. HEV is a virus infecting a range of species, but variants infecting humans can mainly be classified into four genotypes (gts), which differ in geographical distribution, route of transmission and pathogenicity [9]. Gt-1 and gt-2 solely infect humans, are predominant in developing countries and mostly result in self-limiting infections. Nevertheless, mortality in pregnant women reaches up to 25%. Gt-3 and gt-4 infections are mostly zoonotic and are predominant in the industrialized world. Chronic gt-3 and gt-4 infections have been described in immunocompromised patients, which represent an important risk group.

The viral genome translates into three or four proteins, depending on viral genotype (gt). ORF1 encodes a nonstructural polyprotein consisting of eight putative domains and is important for viral replication [10]. Whether this polyprotein is cleaved into several non-structural proteins by either a viral and/or cellular proteases is still a matter of debate. ORF2 and ORF3 are translated from a bicistronic subgenomic replicon. Different forms of ORF2 are produced during the life cycle: an infectious ORF2 that is the component of viral particles, as well as secreted/glycosylated ORF2 and cleaved ORF2 [11,12]. The ORF3 protein is a small phosphoprotein playing a role in multiple cellular processes such as modulating immune responses and is important for viral egress [13,14,15]. A fourth ORF is only observed in gt-1 HEV and is expressed under cellular stress conditions and influences viral replication [16].

The continued effort to define the interactions between the virus and host in general and in particular for HEV, will certainly lead to information about the life cycle of a virus that is far from understood. Previous work on this topic has already led to interesting findings, but overall our knowledge remains limited. To better understand the HEV–host interface as a whole and to compare different genotypes, we generated a protein–protein interaction network by subjecting ORF2 and ORF3 of both gt-1 and gt-3 and ORF4 of gt-1, to two mammalian two-hybrid assays utilizing a prey library of 15,000 proteins. We used bioinformatic databases to analyze these interactions and found significant enriched processes in relation to the respective protein(s). Two proteins from the hit lists, SHARPIN and RNF5, were selected for their interaction with ORF3 and their effect on the ubiquitination status of target proteins involved in the antiviral interferon signaling pathway. Using knockout (KO) cells for the respective proteins, we show they influence HEV-induced interferon (IFN) signaling. Moreover, HEV infection is affected in the KO cells.

## 2. Materials and Methods

### 2.1. High Throughput Screens

The MAPPIT and KISS bait were constructed as follows: the coding sequences of both gt-1 ORF2-4 were amplified from a fecal sample of a HEV Sar-55-infected liver-chimeric mouse, and gt-3 ORF2-3 were amplified from the gt-3 Kernow-C1 passage 6 plasmid. These were fused to the EpoR-LR-F3 chimeric receptor in the pSEL (+2L) vector via SalI-NotI restriction cloning (MAPPIT) or N-terminally to a fragment of human TYK2 in the pSVSport vector via EcoRI/MfeI-NotI restriction cloning (KISS). Primers are listed in Table 1. The obtained products were sequence-verified. The construction of prey constructs and the reverse transfection method was performed as described previously [2,3,17]. High-throughput MAPPIT and KISS screens were performed in microarray and retested in luciferase assay as described previously, using a prey library generated using the hORFeome v8.1 and ORFeome Collaboration clones (Center for Cancer Systems Biology, Boston, MA, USA) [17,18,19,20].

### 2.2. PPI Analysis

Jvenn was used to create Venn diagrams of overlapping proteins [21].

The Database for Annotation, Visualization and Integrated Discovery (DAVID) was used to perform a functional annotation analysis regarding the different GO terms (biological process, molecular function, cellular compartment). DAVID’s modified Fisher Exact *p*-value was set to 0.05, as a *p*-value equal or smaller to this number is considered as strongly enriched in the annotation category. Additionally, functional annotation clustering for the terms Functional Annotation and Gene ontology was performed with default settings and a medium classification stringency.

### 2.3. CRISPR-Cas9 Knockout of SHARPIN and RNF5

Single-guide RNAs (sgRNA) targeting the DNA sequence of SHARPIN and RNF5 were designed using the CRISPR gRNA design tool of Atum https://www.atum.bio/eCommerce/cas9/input (accessed on March–April 2020). Oligo pairs encoding the guide sequences were annealed and ligated into the sgRNA expressing plasmid, pX458 (kindly provided by Feng Zhang, Addgene plasmid #48138) [22]. The sequences of the oligo pairs are represented in Table 2. PLC3 cells were transfected with the CRISPR plasmids using lipofectamine 2000 (Thermo Fisher Scientific, Waltham, MA, USA). In brief, 3 × 10^5^ cells were seeded per well in 6-well plates and transfected with 2500 ng DNA and 12.5 µL lipofectamine. Two days after transfection, single-cell sorting of GFP+ cells was performed using a BD FACS Aria III cell sorter (Becton Dickinson, NJ, USA). Clonal cell populations were expanded and checked for absence of protein expression by Western blot.

### 2.4. HEV ORF3 Transfection and IFN qRT-PCR Assays

An HEV ORF3 plasmid was constructed as follows. The ORF3 sequence was amplified from the pSK-E2 cDNA clone with following primers: 5′-aagcttaccatgaataacatgtcttttgctgcgcccatgggttc-3′ and 5′-gcggccgcttagcggcgcggccccag-3′. Purified PCR products were cloned into pcDNA3.1 using HindIII and NotI restriction sites and resulting construct was verified by sanger sequencing.

PLC3^WT^ and PLC3^SHARPIN-KO^ and PLC3^RNF5-KO^ were maintained at 37 °C and 5% CO_2_ in Dulbecco’s Modified Eagle’s Medium (DMEM) (Thermo Fisher Scientific, Waltham, MA, USA) supplemented with 10% inactivated FCS (Biowest, Nuaillé, France), 2 mM L-glutamine (Thermo Fisher Scientific, Waltham, MA, USA), 1% non-essential amino acids (Thermo Fisher Scientific, Waltham, MA, USA), 100 U/mL of penicillin and 100 µg/mL streptomycin (Thermo Fisher Scientific, Waltham, MA, USA). The ORF3 expressing plasmid or mock control were transfected using Lipofectamine 2000 (Thermo Fisher Scientific, Waltham, MA, USA), with a final amount of 2500 ng DNA and 9 µL lipofectamine/well. Tested conditions were performed in duplicate. Two days after MOCK or ORF3 transfection, cells were transfected with poly(I:C) (Invivogen, San Diego, CA, USA) to stimulate the RLR pathway as described before [14]. The end concentration of poly(I:C) used was 1 µg/mL. Sixteen hours post stimulation, cells were lysed for Western blot analysis and RNA extraction for downstream RT-qPCR. RNA was extracted using RNeasy plus mini kit (Qiagen, Hilden, Germany).

### 2.5. HEV Infectious Virus Production

The gt-3 HEV Kernow-C1 Passage 6 (p6) plasmid (GenBank accession number JQ679013) was linearized with MluI (New England Biolabs, Ipswich, MA, USA). Capped RNA was produced either using T7 RiboMAX Express Large Scale RNA production (Promega, Madison, WI, USA), followed by a ScriptCap m7G capping system (Cellscript, Madison, WI, USA) or using a T7 mMESSAGE mMACHINE kit (Thermo Fisher Scientific, Waltham, MA, USA). Ten micrograms of capped RNA was electroporated into 9 × 10^6^ PLC3 cells using a Gene Pulser (Bio-Rad, Hercules, CA, USA). Viral particles were harvested from centrifuged and filtered (0.22 µm) supernatant, and concentrated using Amicon ultra centrifugal Filters (100 kDa) (Merck, Darmstadt, Germany).

### 2.6. HEV Infection and HEV ORF2 Immunofluorescence Staining

PLC3^WT^, PLC3^SHARPIN-KO^ and PLC3^RNF5-KO^ were seeded in black 96-well plates and exposed to a dilution of HEV preparation with an MOI of 22,000, defined as 1 IU/cell, and IU (International unit) was quantified by qPCR as described previously [23]. To determine the infectivity titer, cells were fixed by methanol, followed by incubation with 0.5% Triton X-100 and blocking with PBS containing 10% goat serum. HEV ORF2 staining was performed using a mouse ORF2-specific monoclonal antibody (1E6, Merck, Darmstadt, Germany) in combination with an Alexa Fluor 488-conjugated goat anti-mouse antibody. Nuclei were stained with DAPI (4′,6-diamidino-2-phenylindole). Microscopy was performed using a Leica TSC-SPE confocal microscope (10× or 20× objective) (Wetzlar, Germany). Infectivity of the respective well was calculated as a percentage with automated cell counting as well as ORF2 fluorescence with ImageJ software (v1.54d).

### 2.7. SDS-PAGE and Western Blot

Cells were lysed with RIPA Lysis and Extraction buffer (Thermo Fisher Scientific, Waltham, MA, USA), supplemented with Halt Protease and Phosphatase Inhibitor Cocktail (Thermo Fisher Scientific, Waltham, MA, USA). Lysates were denatured in the presence of Bolt LDS sample buffer and Bolt Reducing agent (both Thermo Fisher Scientific, Waltham, MA, USA) at 70 °C for 10 min. Samples were subjected to 12% SDS-PAGE and transferred to a polyvinylidene difluoride membrane (PVDF, 0.2 µm pore size; Thermo Fisher Scientific, Waltham, MA, USA). Detection of the targeted proteins was performed by specific antibodies and corresponding horseradish peroxidase-conjugated secondary antibodies (Table 3). Immunoblots were incubated with SuperSignal™ West Femto Maximum Sensitivity Substrate kit (Thermo Fisher Scientific, Waltham, MA, USA) and imaged on an ImageQuant LAS4000 chemiluminescent imaging system (GE Healthcare, Diegem, Belgium).

### 2.8. Reverse Transcription Quantitative PCR (RT-qPCR)

Total RNA was extracted from cultured cells using the RNeasy plus mini kit (Qiagen, Hilden, Germany). cDNA synthesis was performed with SuperScript III reverse transcriptase (Thermo Fisher Scientific, Waltham, MA, USA). Target and reference gene transcripts were detected using pre-designed Taqman primer-probe assays (Thermo Fisher Scientific, Waltham, MA, USA). All used assays are listed in Table 4. cDNA was diluted 2.5× (target genes) or 10× (reference genes) and 2 µL was used as input for qPCR reaction using TaqMan Fast Advanced Master mix (Thermo Fisher Scientific, Waltham, MA, USA). Cycles of quantification were generated on a LightCycler 480 (Roche, Bazel, Switzerland) using the second-derivative maximum method. Calibrated normalized relative quantities (CNRQs) were calculated for each target gene with qBasePlus Software v3.1 (CellCarta, Montreal, QC, CA), using the most stable reference genes as selected by GeNorm analysis in the qBasePlus Software.

### 2.9. Statistical Analyses

Statistical analysis was performed using GraphPad Prism 8.0.1 software. Statistical significance of differences between groups was evaluated with *t*-tests.

## 3. Results

### 3.1. MAPPIT and KISS Are Functional Tools to Study HEV–Host Protein–protein Interactions

ORF2 and ORF3 from both gt-1 and gt-3 and ORF4 (gt-1) were used as bait in the two related assays MAPPIT and KISS.

**Figure 1:**
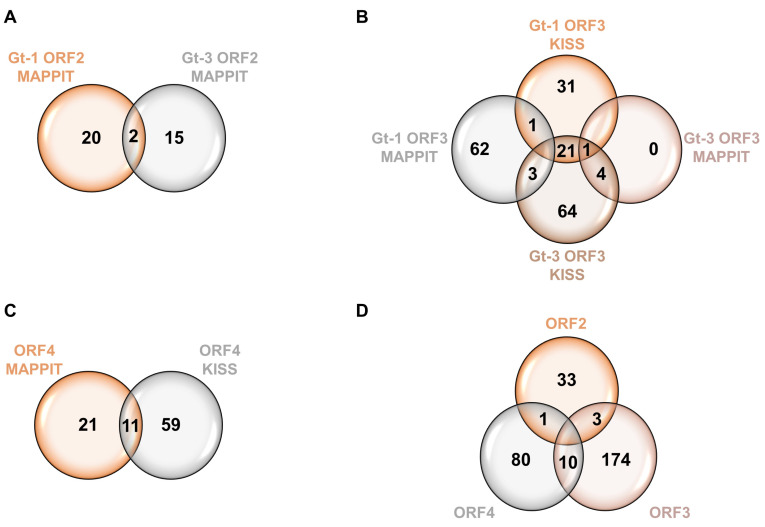
Venn diagrams depicting HEV ORF interactors identified by MAPPIT and KISS analyses. (**A**) Interactors of the ORF2 protein identified by MAPPIT analyses. (**B**) Interactors of the ORF3 protein identified by both MAPPIT and KISS analyses. (**C**) Interactors of ORF4 protein identified by both MAPPIT and KISS analyses. (**D**) Venn diagram shows distinct and overlapping interacting proteins of all HEV proteins analyzed.

Using this approach, we had four constructs for ORF2: gt-1 ORF2 MAPPIT, gt-1 ORF2 KISS, gt-3 ORF2 MAPPIT and gt-3 ORF2 KISS. Microarrays were performed and revealed 37 hits in total for this protein (Figure 1A). These were uncovered solely with the MAPPIT constructs, as the KISS assay failed to reveal any interactions. Comparing both genotypes, we found that two proteins were shared, specifically CA12 and ADCY3 (Figure 1A) (Appendix A Table A1).

Likewise, four constructs were created for ORF3: gt-1 ORF3 MAPPIT, gt-1 ORF3 KISS, gt-3 ORF3 MAPPIT and gt-3 ORF3 KISS, revealing 187 hits in total (Figure 1B). Most hits were shared between the KISS assay of the two different genotypes, and this initial screening showed an overlap of 25 hits between gt-1 and gt-3 (Figure 1B) (Appendix A Table A1).

For ORF4, combining MAPPIT and KISS revealed 91 hits, of which 11 are shared between the two assays (Figure 1C) (Appendix A Table A1).

To obtain a general overview of all the HEV hits discovered with MAPPIT and KISS, we compiled all the different hits per HEV protein, irrespective of the used method or genotype, and looked whether there are particular hits shared between HEV ORF2, ORF3 and ORF4, as HEV proteins often interact with each other during the viral life cycle. Following this approach, the Venn diagram shows that a substantial amount of hits were shared between both ORF3 and ORF4, STUB1 was shared between ORF2 and ORF4; and ORF2 and ORF3 shared FTL, TMEM154 and FCGR2A (Figure 1D) (Appendix A Table A1).

### 3.2. Functional Annotation Clustering for the HEV Proteins

To gain a comprehensive overview of the hits and relate biological relevance to those identified, we first performed gene ontology (GO) annotation for each protein with the DAVID bioinformatics tool.

**Figure 2:**
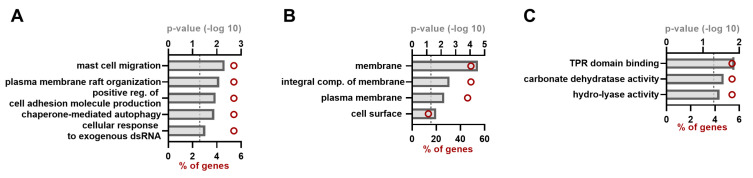
Gene ontology (GO) annotation of the ORF2 hit list. GO annotation for the ORF2 interacting proteins of both gt-1 and gt-3 as identified by MAPPIT analysis; different categories are depicted: biological process (**A**), cellular compartment (**B**) and molecular function (**C**). Red circles indicate the number of proteins belonging to each term and shown as percentage of the total ORF2 hit list (=37). *p*-value was set to equal to or smaller than 0.05 (indicated by dashed line).

For ORF2, five significant clusters could be observed for biological processes (BP) (Figure 2A, Appendix A Table A2), all containing two proteins or 5.4% of the ORF2 dataset. From the cellular compartment (CC) clusters, it is obvious that almost half of the amount of hits are associated with the (plasma) membrane, and about 14% is associated with the cell surface (Figure 2B, Appendix A Table A2). Three clusters could be observed regarding the molecular function (MF) annotation, being TPR-domain binding, carbonate dehydratase activity and hydro-lyase activity, which all contained two proteins or 5.4% of the dataset (Figure 2B, Appendix A Table A2).

**Figure 3:**
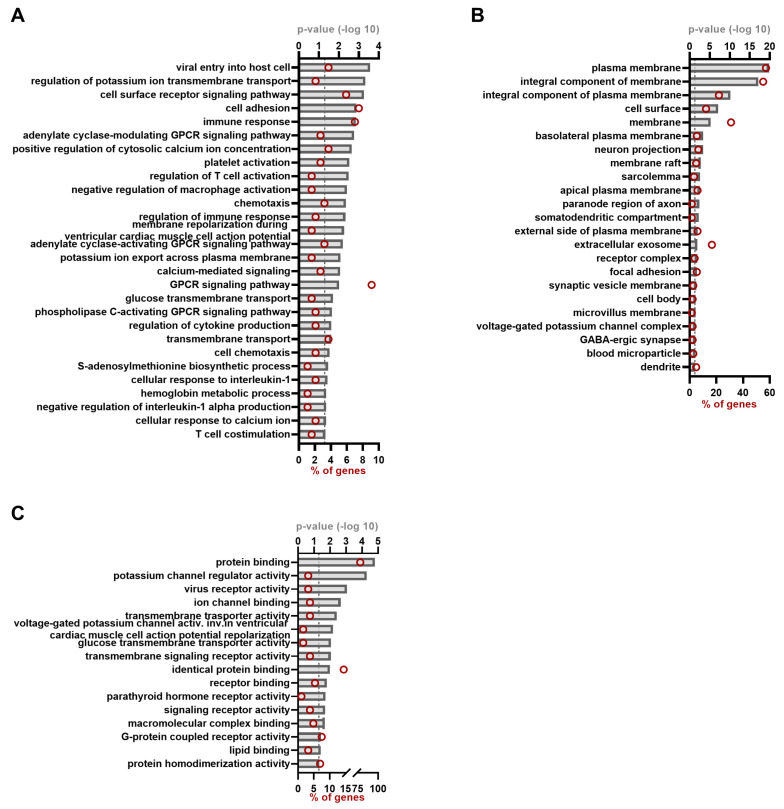
Gene ontology annotation of the ORF3 hit list. GO annotation for the ORF3 interacting proteins of both gt-1 and gt-3 as identified by MAPPIT and KISS analyses. Different categories are depicted: biological process (**A**), cellular compartment (**B**) and molecular function (**C**). Red circles indicate the number of proteins belonging to each term and shown as percentage of the total ORF3 hit list (=187). *p*-value was set to equal to or smaller than 0.05 (indicated by dashed line).

For ORF3, 28 BP clusters were observed, with among them processes such as viral entry into host cells, transmembrane transport, GPCR signaling and immune response (Figure 3A, Appendix A Table A3). As with the ORF2 hits, a high proportion of the total ORF3 hit list reside in membranes or plasma membranes (Figure 3B, Appendix A Table A3).

**Figure 4:**
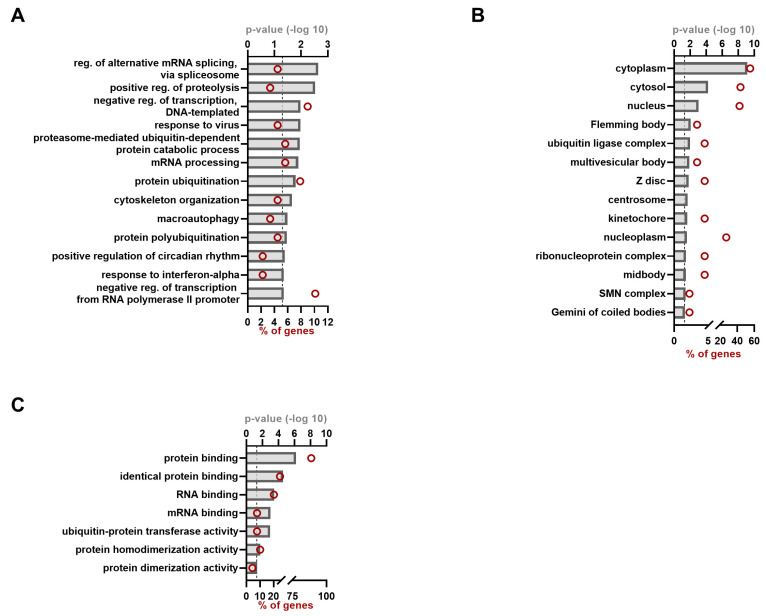
Gene ontology annotation of the ORF4 hit list. GO annotation for the gt-1 ORF4 interacting proteins as identified by MAPPIT and KISS analyses, different categories are depicted: biological process (**A**), cellular compartment (**B**) and molecular function (**C**). Red circles indicate the number of proteins belonging to each term and shown as percentage of the total ORF4 hit list (=91). *p*-value was set to equal to or smaller than 0.05 (indicated by dashed line).

For ORF4, 13 BP clusters could be observed with regulation of alternative mRNA splicing, with the positive regulation of proteolysis and negative regulation of transcription among the most significant ones (Figure 4A, Appendix A Table A4). More than half of the proteins are clustered in the cellular compartment cytoplasm, but a great deal of proteins also cluster to the nucleus. Regarding the MF ontology, 88% of the hits have protein-binding capacities, and about 15% are (m)RNA-binding proteins (Figure 4C, Appendix A Table A4).

As mentioned in the previous section, HEV proteins often interact with each other and therefore, we were interested to see if the identified candidate interactors would cluster together in certain (upregulated) GO terms. Functional annotation clustering of the total HEV hit list was performed in DAVID and revealed 22 clusters (Appendix A Table A5). We could again observe that most of the proteins are observed in a membrane cluster. The second most enriched cluster involved proteins important for viral entry or receptor activity. Proteins related to GPCR signaling were again observed. Cluster 5 and 6 relate to ion channel terms and may be related to the function of ORF3 as an ion channel. Inflammatory response and immunity also emerged and molecular functions relating to post-translational modifications such as protein phosphorylation and ubiquitination are also present.

Additionally, we also performed a search for hits that have previously been described in the context of viral infections (Appendix A Table A6). Specifically, we found that 15 of the 37 ORF2-interacting proteins identified with MAPPIT and KISS are in some way involved in viral infections. Likewise, 108 of the 187 identified ORF3-interacting host proteins and 33 ORF4-interacting protein hits have previously been described to play a role in viral infections.

To further scrutinize the proteins, we focused on hits that were identified either for both genotypes, or in both assays, or shared among HEV proteins. The selection was further narrowed down by investigating if potential hits are known to be important in viral infections (Appendix A Table A6). One of the hits we observed in the clusters of protein ubiquitination is SHARPIN or SHANK-Associated RH Domain Interactor. In our analysis, we observed this as a hit of gt-1 ORF3, identified by both MAPPIT and KISS assay, and as a hit of ORF4, also identified by both MAPPIT and KISS assay. We speculated that gt-3 ORF3 could also bind SHARPIN. Therefore, we performed a retest of the gt-3 ORF3 in the KISS configuration, as this proved the most effective for this genotype in our initial analysis. Using this approach, we could confirm that gt-3 ORF3 also bound SHARPIN (Table 5).

In the same cluster of protein ubiquitination, we identified the Ring Finger Protein 5 (RNF5). During our analysis, this appeared a candidate hit of gt-1 ORF3. Along with the experiments we performed for SHARPIN, we speculated that gt-3 ORF3 could also bind RNF5. Upon retest, we could indeed confirm the interaction of gt-3 ORF3 with RNF5 (Table 5).

### 3.3. SHARPIN Affects the Induction of Interferon upon ORF3 Transfection

SHARPIN forms the linear ubiquitin chain assembly complex (LUBAC) which mainly targets proteins in the Retinoic acid-Inducible Gene I (RIG-I)-like receptor (RLR)-Mitochondrial Antiviral Signaling Protein (MAVS) Pathway.

**Figure 5:**
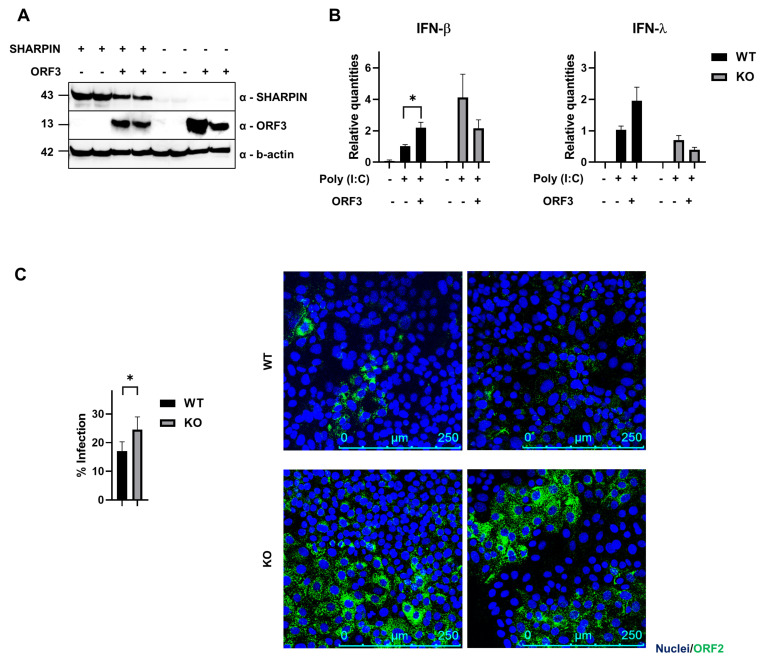
SHARPIN affects the induction of IFN response. (**A**) PLC3^WT^ and PLC3^SHARPIN-KO^ cells (indicated, respectively, with + and −), are mock- or HEV ORF3-transfected (indicated, respectively, with − and +). Successful knockout and ORF3 transfection verified by Western blot. B-actin is displayed as a control. Two replicates of each condition are displayed. (**B**) PLC3^WT^ and PLC3^SHARPIN-KO^ cells were mock- or HEV ORF3-transfected and two days later transfected with poly (I:C). IFN-β (left) and IFN-λ mRNA levels measured by qRT-PCR. Transcripts normalized to reference genes (ATP5B, CYC1, YWHAZ, HPRT1, RPL30) and scaled to PLC3^WT^—MOCK (ORF3-). (**C**) PLC3^WT^ and PLC3^SHARPIN-KO^ cells infected with HEV (MOI 22000) and infection levels analyzed 6 days later by HEV ORF2 immunostaining. Representative confocal images on the right. Nuclei in blue and ORF2 in green. Results are represented as mean ± SEM compiled from three independent experiments. Statistical significance calculated by (multiple) *t*-test. Differences were considered as statistically significant when *p* < 0.05 (*).

To understand the roles of this protein in the context of HEV infection, a CRISPR-Cas9 knockout (KO) cell line was generated (Figure 5A). We used PLC3 cells, a subclone of PLC/PRF/5, that are permissive to HEV replication [11].

To understand a potential interaction of this protein with ORF3, we expressed this protein in PLC3^WT^ and PLC3^SHARPIN-KO^ (Figure 5A) and investigated its effect on the induction of IFN, since LUBAC has been shown to regulate signaling downstream of TLR3, RIG-I and MDA5. According to what was previously published [14], we could show that ORF3 also boosted poly (I:C) mediated-type I IFN induction in PLC3 cells. Viral protein-transfected cells showed a response twice as high as the mock control (*p* = 0.017), while unstimulated cells had an absent or only very minimal response (Figure 5B, left panel). Strikingly, in the SHARPIN KO, the ORF3-induced enhancement of type I IFN was abolished (*p* = 0.24) and rather tended to show an inverse effect (Figure 5B, left panel). Likewise, we investigated the type III IFN response. Although the trend seemed to be the same as for type I IFN, we could not show a significant induction of type III IFN upon ORF3 addition in PLC3^WT^ cells (*p* = 0.13) (Figure 5B, right panel). We next wondered if these changes in interferon response could have an impact on HEV infection. To this end, WT and SHARPIN-KO cells were infected and the level of infection was measured by immunostaining 6 days later. Strikingly, infection increased from 17% in WT cells to 25% in the SHARPIN-KO cells (*p* = 0.0357) (Figure 5C). Together, these results show SHARPIN has an effect on the ORF3-mediated IFN induction, while infection is stimulated in the KO cells.

### 3.4. RNF5 Interferes with IFN Induction upon ORF3 Transfection

**Figure 6:**
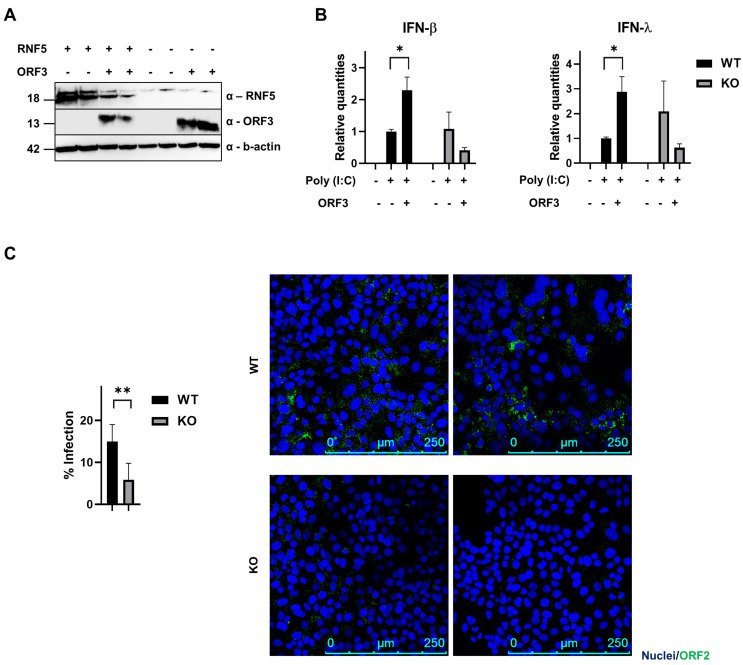
RNF5 affects the induction of IFN response. (**A**) PLC3^WT^ and PLC3^RNF5-KO^ cells (indicated, respectively, with + and −), are mock- or HEV ORF3-transfected (indicated, respectively, with − and +). Successful knockout and ORF3 transfection verified by Western blot (upper band in the RNF5 blot is aspecific). B-actin is displayed as a control. Two replicates of each condition are displayed. (**B**) PLC3^WT^ and PLC3^RNF5-KO^ cells were mock- or HEVORF3-transfected and two days later transfected with poly (I:C). IFN-β (left) and IFN-λ mRNA levels measured by qRT-PCR. Transcripts normalized to reference genes (ATP5B, CYC1 and YWHAZ) and scaled to PLC3^WT^—MOCK (ORF3-). (**C**) PLC3^WT^ and PLC3^RNF5-KO^ cells infected with HEV (MOI 22,000) and infection levels analyzed 6 days later by HEV ORF2 immunostaining. Representative confocal images on the right. Nuclei in blue and ORF2 in green. Results represented as mean ± SEM from three independent experiments. Statistical significance calculated by (multiple) *t*-test. The level of significance is indicated with *p* < 0.01 (**) or *p* < 0.05 (*).

The Ring Finger Protein 5 (RNF5) is a membrane-bound ubiquitin ligase that is known to inhibit IFN type I in the context of virus infections. To investigate a potential effect of RNF5 in the HEV life cycle by interacting with ORF3, we performed a similar experiments as for SHARPIN above. PLC3^RNF5-KO^ cells were generated and successful KO was verified by Western blot; ORF3 was expressed in these cells and compared to PLC3^WT^ (Figure 6A). ORF3 enhanced the type I IFN response upon poly I:C stimulation (*p* = 0.022), but this was not observed in the RNF5-KO cells. Moreover, the trend seems to be that IFN-β diminishes upon ORF3 transfection in these cells (*p* = 0.28) (Figure 6B, left panel). For type III IFN responses, we observed the same trend: there was an increase upon ORF3 transfection in WT cells (*p* = 0.023), which was not observed in the KO cells (*p* = 0.26) (Figure 6B, right panel).

Furthermore, we checked if there is an influence on HEV infection in PLC3^RNF5-KO^ cells compared to PLC3^WT^. Cells were infected and the amount of infection was measured by immunostaining 6 days later. Infection reduced from 15% in the WT population to 6% in the KO population (Figure 6C). These results show that RNF5 interferes with the ORF3increased IFN response and influences HEV infection.

## 4. Discussion

In this study, we used two PPI methods to identify interactions of the HEV ORF2-4 to aid in the urgent need to better understand the HEV pathogenesis, life cycle and virus–host interactions. Some HEV PPI studies have been performed in the past, most of them using yeast-two-hybrid and affinity purification in combination with mass spectrometry [24,25,26,27,28,29]. One method can only detect a certain fraction of all PPIs, illustrating that in order to comprehensively map the interactome of one particular protein of interest, it is important to use different methods and consider them as complementary rather than as independent assays [30]. The advantage of the MAPPIT and KISS screenings used in this study is their mammalian background and high-throughput capability [2,3,19]. We validated this approach for HEV and identified 37 candidate interactors for ORF2, 187 for ORF3 and 91 for ORF4. We did not investigate ORF1 due to the controversy and complexity about the processing of this protein, and the studies conducted so far with this protein have always used the individual domains as bait [31,32]. Additionally, ORF1 interacts with JAK2 [32], which means it could bind the JAK2 proteins that are constitutively associated with the chimeric MAPPIT receptor, posing an additional difficulty.

For ORF2, our candidate interactors were identified only in the MAPPIT configuration. In initial tests of the KISS screening, positive controls that bind the Tyk2 domain did not result in acceptable signals and a pre-screen with known ORF2 interactions did not reveal any hits.It is unclear why this happened. It could point to a technical issue of the assay but it is more likely that it is attributed to the nature of the HEV ORF2 protein, as this is the only one of the HEV proteins that failed. In MAPPIT, the bait moiety is tethered to the plasma membrane, whereas in KISS, the bait can shuttle in the cytosol. It is known that ORF2 translocates to the nucleus and reticular compartments; therefore, it is possible that by residing in these subcellular compartments, the construct is hidden for potential interactions with prey proteins in the cytosol [33]. Before screening, we anticipated that the subcellular localization of HEV proteins could impede some analyses, especially since it is known, for example, that replication of viruses can induce membrane rearrangements to shield for immune responses [34]. For this reason, we decided to perform screenings with both methods.

In our 37 identified ORF2 hits, we could confirm four previously described interactions of this HEV protein: HSP90, TMBIM4, FTL and CYB5A [24,26]. Only two hits were shared between gt-1 and gt-3 in our assay, being CA12 and ADCY3 (Figure 1A); the former protein is part of the zinc metalloenzymes catalyzing the reversible hydration of carbon dioxide, whereas ADCY3 catalyzes the formation of the secondary messenger cyclic adenosine monophosphate (cAMP). Gene ontology annotation analysis revealed that most of the identified ORF2 hits are membrane-associated proteins, which is in agreement with what was published in another ORF2 screen using Y2H [26]. Looking at the biological processes, there were two proteins that were often observed: FLOT1 and COLEC12 (Figure 2A). FLOT1 is an integral membrane component of caveolae and plays a role in vesicle trafficking and cell morphology. Additionally, flotillin-dependent endocytosis is an alternative pathway, independent of clathrin or dynamin that could in theory be used by viruses. However, mechanisms remain unclear and many studies report no involvement of the flotillin in virus entry, including for hepatitis C virus or hepatitis A virus (HAV) [35,36,37]. However, FLOT1 is present in HAV exosomes [38], which might also be the case for HEV, and a potential interaction with FLOT1 might be of importance during membrane fusion in the process of viral release [39]. COLEC12 is a member of the C-lectin family: it is a scavenger receptor that may function as pattern recognition molecule (PRM) by binding carbohydrate antigens on microorganisms (particularly bacteria) to facilitate their removal. However, no clear studies have yet confirmed this role in the context of viral infection, except one study showing no binding of the SARS-CoV-2 spike to COLEC12, while it did bind other related humoral pattern recognition molecules [40]. The expression of COLEC-12 in human hepatocytes was confirmed in a study involving the hepatitis C virus, but the role of the protein was not further investigated [41].

For ORF3, we confirmed the previously described interactions with TSG101, NAT1, MAT1A, HSPA8, HPX, FTL, ALDOB, C151, FGB, APOH and AMBP and an additional 176 hits that were not previously described. Strikingly, the most significant biological process we observed was the one containing proteins involved in ‘viral entry into host cells’ (Figure 3A). EFNB2 is a receptor for Hendra and Nipah virus [42], CD4 for human immunodeficiency virus [43], CXADR is the coxsackievirus and adenovirus receptor [44], and BSG has been shown to be involved in infection of measles and human cytomegalovirus [45,46]. AGTR1 also plays a role during SARS-CoV-2 endocytosis and NECTIN-4 is a receptor for measles virus [47,48]. These proteins mostly bind a viral protein to mediate viral entry, so it is intriguing why ORF3 is observed as an interacting partner of these proteins. Only HAVCR1 is a phosphatidylserine receptor that mediates the entry of a variety of viruses that acquire PS in their viral envelope through a process called ‘apoptotic mimicry’ [49]. We very recently studied the role of this protein in the entry of HEV and could demonstrate the involvement of PS acquired in the enveloped form of the HEV virions [50]. The observation of all these viral receptors might indicate that the role of ORF3 during entry of enveloped hepatitis E virus is of more importance than we think and should be further studied. We also observed some BPs and MFs related to ion transmembrane transport (Figure 3), which could be related to the function of ORF3 as a functional ion channel in the release of virions [13]. We also observed some BPs regarding the immune response. For ORF3, it was previously described that it can interfere with cellular host defenses and its interferon-induced effectors, sometimes by inhibiting IFN induction, or by induction of IFN [14,51,52]. Most of the proteins here were also membrane proteins (Figure 3B). Intriguingly, we observed an annotation term of extracellular exosome, containing 31 proteins. It is well known that ORF3 uses the exosome pathway, a process orchestrated by the interaction of ORF3 and TSG101 to dock virions in the multivesicular bodies leading to viral release [53].

For ORF4, only two previous studies have been performed to screen for interacting proteins [16,54]. The hits we observed during our MAPPIT and KISS analysis were not reported before. We observed that ORF3 and ORF4 shared 10 protein hits. Previously, it was reported that ORF4 forms a protein complex with the RdRp, helicase and X, of which the assembly is inhibited by interaction with ORF3 [16]. It is possible ORF3 and ORF4 form another complex together with host proteins to exert this and/or other functions. Interestingly, TSG101 seems to be one of the proteins that is shared between ORF3 and ORF4. Another multivesicular body protein, CHMP4C, interacted with ORF4, illustrating it might be worthwhile to study this interaction in the context of gt-1 virus assembly and release. For ORF4 BPs, we could also observe proteins related to virus responses. Both ADAR and PRKRA (PACT), part of the RNA sensing pathway, were observed to interact with ORF4. ADAR has both pro- and antiviral activities, one of them by limiting the amount of dsRNA and thereby inhibiting PKR activation. Furthermore, PKR is activated by PACT and its activation inhibits cellular mRNA translation through the phosphorylation of EIF2A [55,56]. It is intriguing that ORF4 binds both these proteins. It was also reported that ORF4 is a target of the proteasome due to ubiquitination of Lysine at the 51st amino acid position [16]. Several proteins in our hit list were linked to a (poly)ubiquitination status (Figure 4), most of them are (part of) E3 ubiquitin ligases (complex). FBXW11 and BTRC are even part of the same complex. It would be interesting to see if one of the observed interactors is able to ligate ubiquitination to ORF4. On the other hand, these proteins often ubiquitinate target proteins involved in innate defense pathways such as NF-kB pathways. Additionally, SHARPIN and TRAF1 were also identified as ORF4 interactors, and previous work illustrated that TRAF1 is a target of the LUBAC complex, consisting of HOIP, HOIL-1L and SHARPIN, to enhance NF-kB activation. On the contrary, it was also reported that TRAF1 sequesters LUBAC to limit ubiquitination of another target NEMO, thereby limiting NF-kB activation [57]. It remains to be elucidated if a similar process could be observed upon ORF4 interaction.

Many of the proteins we discovered as HEV-interacting proteins were already described to be implicated in other viral infections (Appendix A Table A6). This clearly indicates the relevance of the interactome we generated in this work. For an abundance of these host proteins, this role was linked to an increased or decreased expression upon viral infection or relates to data generated by other PPI-screens. The specific role that the identified host protein played in the viral life cycle was not necessarily further investigated. For other host proteins, including the already-mentioned HAVCR1, ADAR, EFNB2, CXADR and NECTIN4, their role has already been more thoroughly studied, making a potential relationship to the HEV life cycle more easy. A disadvantage of the here-applied PPI methods, but by extension of similar approaches such as yeast-two-hybrid, is that they rely on interactions between proteins that are artificially brought together in the same cell by ectopic expression. Confirming biological relevance of the uncovered interactions is therefore of the utmost importance. We selected SHARPIN and RNF5 for further characterization.

Initially, we only observed an interaction of both SHARPIN and RNF5 with gt-1 ORF3. By retesting the constructs in a second MAPPIT and KISS screening experiment, we could confirm the interaction for both ORF3 (Table 5). Again this illustrates that additional hits may be found upon repetition of a certain PPI method. That we confirmed the interaction with gt-3 for both SHARPIN and RNF5 is not unusual: amino acid sequence homology between the used gt-1 Sar55 strain and gt-3 Kernow-C1 p6 strain is more than 80%, but gt-specific regions exist. Similarly, Geng et al., identified 32 shared human proteins interacting with ORF3 from human isolates of gt-1 and gt-4, and with ORF3 from a gt-3 rabbit isolate [28].

SHARPIN, together with HOIL-1 and HOIP, forms LUBAC, the only complex known so far able to polyubiquitinate target proteins in a linear fashion. Studies regarding this complex in viral infections have led to opposing data. For Sendai virus (SeV) and Vesicular stomatitis virus (VSV), it was shown that LUBAC inhibits virus-induced interferon, whereas a study using norovirus showed that parts of the LUBAC complex are essential for the induction of both type I and type III IFN [58,59,60]. Here, we also investigated both the type I and type III IFN response. In addition, it is known that HEV can induce both types as well [61,62,63]. In agreement with the previous literature, we could confirm that ORF3 enhances poly (I:C)-mediated type I IFN induction 1.9–2.3 fold compared to the mock control (Figure 5B and Figure 6B) [14]. Remarkably, when PLC3^SHARPIN-KO^ cells were transfected with ORF3, the poly(I:C)-mediated induction of IFN diminished (Figure 5B). It is thought the ORF3-mediated IFN enhancement is caused by RIG-I activation through K63-linked ubiquitination. Two ubiquitin ligases can mediate this activation: TRIM25 and Riplet [14]. Interestingly, TRIM25 has been described as a target for LUBAC [60]. During SeV, LUBAC suppresses RIG-I ubiquitination and activation by inducing TRIM25 degradation, thereby suppressing subsequent type I IFN production. However, our data would suggest an inverse relationship as absence of SHARPIN/LUBAC might suppress IFN induction upon ORF3 (Figure 5B). Still, since opposing effects of LUBAC (components) have been described for a variety of viruses, the targets may be differentially regulated depending on the virus of interest. These effects were mainly seen upon type I IFN induction, but the trend seems to be the same for type III IFN induction. Interestingly, HEV infection is slightly higher in SHARPIN-KO cells compared to control (Figure 5C), and might be related to the lower level of ORF3-mediated IFN induction in these cells.

Future research should explore which exact proteins belonging to the RLR-MAVS pathway leading to IFN induction are targeted by SHARPIN/LUBAC in the context of HEV. Taking things together, a protein complex between RIG-I, TRIM25, SHARPIN and ORF3 might be a possibility, as mentioned above. Additionally, LUBAC can also target NEMO to attenuate IFN response upon VSV infection, via disruption of the MAVS-TRAF3 complex. The hepatitis B virus induces the protein Parkin to recruit LUBAC to MAVS and disrupt downstream signaling [59,64].

A second candidate interactor of ORF3 that we selected was RNF5, especially because this protein has also been described to influence IFN induction in context of viral infections. For example, the V protein of Newcastle disease virus and PB1 protein of Influenza A virus recruit RNF5 to polyubiquitinate and degrade MAVS, which inhibit type I IFN production [65,66]. Next to MAVS, the protein STING has also been described as a target for RNF5. SeV induces the ubiquitination and degradation of STING by RNF5 affecting IFN signaling [67]. We showed that IFN induction is affected in RNF5-KO cells upon ORF3 transfection (Figure 6B). We also show that HEV infection is affected in these KO cells, with a significantly lower infection level compared to wild-type cells (Figure 6C). It would be interesting to speculate if HEV (ORF3) could interfere with the IFN induction by recruiting RNF5 to its target proteins. One previous study showed that HEV does not induce MAVS degradation; further research should explore whether STING would be a more reasonable target in case of HEV infection [61].

## 5. Conclusions

In conclusion, our data demonstrate that MAPPIT and KISS are valuable tools to probe HEV–host protein interactions. Moreover, our obtained hit list for HEV ORF2-4 provides a wealth of information to find new host factors implicated in the HEV life cycle, which remains far from understood. Confirmation of biological relevance of obtained hits in high-throughput PPI-methods is important. We specifically identified SHARPIN and RNF5 as interactors for both gt-1 and gt-3 ORF3 and showed they interfere with IFN signaling and HEV infection in general.

## Figures and Tables

**Table 1:** PCR primers for amplification of ORF3 for MAPPIT and KISS assays.

Primer Name	Sequence (5′ → 3′)
Gt-1 ORF2 MAPPIT FW	gtcgacgagctccggatccatgcgccctcggcctattttg
Gt-1 ORF2 MAPPIT RV	gcggccgccaaataaactataactcccgagttttacccaccttcatc
Gt-1 ORF2 KISS FW	caattgaccatgcgccctcggcctattttg
Gt-1 ORF2 KISS RV	gcggccgctaactcccgagttttacccaccttcatcttaaggcgctg
Gt-1 ORF3 MAPPIT FW	gtcgacgagctccggatccatgaataacatgtcttttgctgcgcccatg
Gt-1 ORF3 MAPPIT RV	gcggccgcggagcgaccgcggttagc
Gt-1 ORF3 KISS FW	caattgaccatgaataacatgtcttttgctgcgcccatgggttc
Gt-1 ORF3 KISS RV	gcggccgcgcggcgcggccccagctg
Gt-1 ORF4 MAPPIT FW	gtcgacgagctccggatccatgttgcgcggacagcaaatc
Gt-1 ORF4 MAPPIT RV	gcggccgcttagctcacatacatccgcagggcag
Gt-1 ORF4 KISS FW	caattgaccatgttgcgcggacagcaaatc
Gt-1 ORF4 KISS RV	gcggccgcgctcacatacatccgcagggcag
Gt-3 ORF2 MAPPIT FW	gtcgacgagctccggatccaccatgtgccctagggttg
Gt-3 ORF2 MAPPIT RV	gcggccgcttaagactcccgggttttgcctacc
Gt-3 ORF2 KISS FW	caattgaccatgtgccctagggttgttc
Gt-3 ORF2 KISS RV	gcggccgcagactcccgggttttgcctacc
Gt-3 ORF3 MAPPIT FW	gtcgacgagctccggatccaccatgggatcaccatgtgccctagg
Gt-3 ORF3 MAPPIT RV	gcggccgctcaacggcgcagccccagc
Gt-3 ORF3 KISS FW	caattgaccatgggatcaccatgtgccctagggttg
Gt-3 ORF3 KISS RV	gcggccgcacggcgcagccccagctg

Restriction sites are underlined.

**Table 2:** Sequences of oligos used for annealing and cloning into pX548.

Gene Targeted	Oligo Name	Sequence (5′ → 3′)
SHARPIN	SHARPIN_ FW	CACCGCCTAGTCCGAGGTGCCACCG
	SHARPIN_ RV	AAACCGGTGGCACCTCGGACTAGGC
RNF5	RNF5_ FW	CACCGAAGCCCCCGGTATCACCAAA
	RNF5_ RV	AAACTTTGGTGATACCGGGGGCTTC

**Table 3:** List of antibodies used for Western blot.

Antibody		Reference
Primary antibodies	SHARPIN	MAB8100, R&D systems, Mineapolis, MN, USA
	RNF5	PA5-31793, Invitrogen, Waltham, MA, USA
	ORF3	Bs-0212R, Bioss, Woburn, MA, USA
	β-ACTIN	BA3R, Invitrogen, Waltham, MA, USA
Secondary antibodies	Sheep anti-mouse HRP-linked	NA931, Cytiva, Amersham, UK
	Donkey anti-rabbit HRP-linked	NA934, Cytiva, Amersham, UK

**Table 4:** List of predesigned primer/probe pairs used for RT-qPCR.

Gene Name	Assay ID
IFNB1	Hs01077958_s1
IFNL2/3	Hs04193049_gH
HPRT1	Hs99999909_m1
RPL30	Hs00265497_m1
CYC1	Hs00357717_m1
YWHAZ	Hs01122445_g1
ATP5B	Hs00969569_m1

**Table 5:** Overview of the interaction of SHARPIN and RNF5 with gt-1 and gt-3 ORF3 in MAPPIT and KISS.

Construct	Entrez_ID	Value PIB	Value BP	BP/PIB	BP/BIP	Lowest Value (Min BP/PIB and BP/BIP)	SCORE
Gt-1 ORF3 MAPPIT	SHARPIN	1.17	82.09	69.9	61.2	61.2	+
Gt-1 ORF3 KISS	SHARPIN	1857	796,444	429	42.2	42.2	+
Gt-3 ORF3 KISS	SHARPIN	1857	187,953	101.2	25.4	25.4	+
Gt-1 ORF3 KISS	RNF5	3114	250,917	80.6	33.8	33.8	+
Gt-3 ORF3 KISS	RNF5	3114	365,420	117.4	19.4	19.4	+

Results are compiled from the luciferase reporter read-out. The positive score of an interaction is related to the lowest value of BP/PIB and BP/BIP. In MAPPIT, a fold induction of a specific bait–prey interaction (BP) is calculated by dividing the average of EPO stimulated and non-stimulated controls. The obtained value of the specific BP is then compared to a fold induction of a bait with irrelevant prey interaction (BIP) and to a fold induction of an irrelevant bait and prey interaction (PIB). The BIP in this case was 1.34. To score an interaction as positive, the obtained lowest value of PB/BIP and PB/PIB needs to be above a threshold of 9. In KISS, stimulation is not possible and the average value of the bait–prey interaction is divided by the average of the BIP or PIB and needs to be above a threshold of 5. BIP in this case was 7414. The chosen thresholds depend on positive and negative reference sets and in this case relate to a false positivity rate of 2%.

## Data Availability

Supporting data in Appendix A are available on request.

## Appendix A

**Table A1:** Overview of hits in common between MAPPIT and KISS screens.

Screen	Shared Protein Hits
Gt-1 ORF2 MAPPIT|Gt-3 ORF2 MAPPIT	CA12, ADCY3
Gt-1 ORF3 MAPPIT|Gt-1 ORF3 KISS	SHARPIN
Gt-1 ORF3 KISS|Gt-3 ORF3 KISS	TMEM108, FAM171B, MCAM, TREML1, GPRC5D, SPN, MAG, HS1BP3, OPALIN, BSG, ERMAP, BTN3A2, CRB3, NUMB, PVRL4, TMEM184A, PTH1R, VSIG8, SLC7A8, PTGER4, SURF4
Gt-1 ORF3 MAPPIT|Gt-3 ORF3 KISS	CX3CL1, CYBRD1, HAVCR1
Gt-3 ORF3 MAPPIT|Gt-3 ORF3 KISS	NAT1, MAT1A, HSPA8, HPX
Gt-1 ORF3 KISS|Gt-3 ORF3 KISS|Gt-3 MAPPIT	TSG101
ORF4 MAPPIT|ORF4 KISS	SHARPIN, C9orf169, MSI2, C3orf56, CELF5, TRAF1, LPXN, RBFOX1, THAP8, FAM168A, THAP11
ORF2|ORF3	TMEM154, FCGR2A, FTL
ORF3|ORF4	FAM92A1, RBPMS, SHARPIN, SMN2, C9orf169, RCHY1, OLIG1, TSG101, CCDC170, METTL16
ORF2|ORF4	STUB1

**Table A2:** GO analysis of hits identified during MAPPIT and KISS screens against the HEV ORF2 protein.

Annotation Category	GO Term	Gene Name
Biological Process	GO:0097531~mast cell migration	STAT5B, KITLG
	GO:0044857~plasma membrane raft organization	COLEC12, FLOT1
	GO:0060355~positive regulation of cell adhesion molecule production	COLEC12, FLOT1
	GO:0061684~chaperone-mediated autophagy	HSP90AA1, STUB1
	GO:0071360~cellular response to exogenous dsRNA	COLEC12, FLOT1
Cellular Compartment	GO:0016020~membrane	CA12, COLEC12, CYB5A, HSP90AA1, CMTM6, TOMM34, ADCY3, GBA2, EDA2R, TMEM154, KITLG, FUBP3, TMBIM4, CA4, FLOT1, C5ORF60, LGALS8, FTL
	GO:0016021~integral component of Membrane	CA12, COLEC12, CYB5A, CMTM6, TOMM34, NDUFB1, ADCY3, TNFRSF10B, GBA2, EDA2R, TMEM154, KITLG, FCGR2A, TMBIM4, IGDCC3, CA4, C5ORF60, TIGIT
	GO:0005886~plasma membrane	CA12, COLEC12, HSP90AA1, ANXA4, CMTM6, ADCY3, TNFRSF10B, GBA2, EDA2R, KITLG, FCGR2A, RAB42, CAPNS2, IGDCC3, CA4, FLOT1, TIGIT
	GO:0009986~cell surface	TGIT, TNFRSF10B, ANXA4, CA4, HSP90AA1
Molecular Function	GO:0030911~TPR domain binding	HSP90AA1, STUB1
	GO:0004089~carbonate dehydratase activity	CA12, CA4
	GO:0016836~hydro-lyase activity	CA12, CA4

**Table A3:** GO analysis of hits identified during MAPPIT and KISS screens against the HEV ORF3 protein.

Annotation Category	GO Term	Gene Name
Biological Process	GO:0046718~viral entry into host cell	EFNB2, CD4, CXADR, BSG, AGTR1, NECTIN4, HAVCR1
	GO:1901379~regulation of potassium ion transmembrane transport	KCNE1, KCNIP1, KCNIP2, KCNAB3
	GO:0007166~cell surface receptor signaling pathway	SPN, CD4, FCGR2A, ADGRF5, BSG, PTH2R, PTH1R, ADRB2, MS4A12, FCGR2B, IL27RA
	GO:0007155~cell adhesion	FGB, CD151, AMBP, SEMA4D, MCAM, CD99L2, CX3CL1, EFNB2, EFNB1, MAG, CD4, BSG, SSPN, NECTIN4
	GO:0006955~immune response	PTGER4, SEMA4D, IL1R2, CXCR6, CX3CL1, IL27RA, SPN, CD4, CD7, FCGR2B, ICOS, CAMP, CMKLR1
	GO:0007188~adenylate cyclase- modulating G-protein coupled receptor signaling pathway	PTGER4, FPR1, PTH2R, PTH1R, ADRB2
	GO:0007204~positive regulation of cytosolic calcium ion concentration	PTGER4, FPR1, AGTR1, LPAR1, S1PR3, CXCR6, CMKLR1
	GO:0030168~platelet activation	FGB, DGKG, TREML1, ENTPD2, PDPN
	GO:0050863~regulation of T cell activation	SPN, CD4, TREX1
	GO:0043031~negative regulation of macrophage activation	ADGRF5, FCGR2B, CD200
	GO:0006935~chemotaxis	SPN, FPR1, CXCR6, PLP2, CX3CL1, CMKLR1
	GO:0050776~regulation of immune response	SPN, FCGR2A, FCGR2B, CD200
	GO:0098915~membrane repolarization during ventricular cardiac muscle cell action potential	KCNE1, KCNE4, SCN2B
	GO:0007189~adenylate cyclase-activating G-protein coupled receptor signaling pathway	PTGER4, ADGRF5, LPAR1, S1PR3, PTH1R, ADRB2
	GO:0097623~potassium ion export across plasma membrane	KCNE1, KCNIP2, KCNE4
	GO:0019722~calcium-mediated signaling	TREML1, CD4, AGTR1, CXCR6, MCOLN1
	GO:0007186~G-protein coupled receptor signaling pathway	ENTPD2, FZD7, LPAR1, FPR1, MRGPRF, GPBAR1, PTH2R, CXCR6, PTH1R, CX3CL1, GPR173, ADGRF5, AGTR1, APLNR, S1PR3, GPRC5D, CMKLR1
	GO:1904659~glucose transmembrane transport	SLC2A12, SLC2A5, SLC2A6
	GO:0007200~phospholipase C-activating G-protein coupled receptor signaling pathway	FPR1, AGTR1, PTH1R, CMKLR1
	GO:0001817~regulation of cytokine Production	BTN3A3, BTN3A2, ERMAP, LITAF
	GO:0055085~transmembrane transport	SLC44A5, SLCO1B1, GJA3, FXYD2, CYBRD1, RNF5, SLC2A6
	GO:0060326~cell chemotaxis	AGTR1, LPAR1, CXCR6, CX3CL1
	GO:0006556~S-adenosylmethionine biosynthetic process	MAT1A, METTL16
	GO:0071347~cellular response to interleukin-1	FGB, MMP2, CAMP, CX3CL1
	GO:0020027~hemoglobin metabolic process	HPX, AMBP
	GO:0032690~negative regulation of interleukin-1 alpha production	IL1R2, CX3CL1
	GO:0071277~cellular response to calcium ion	SYT5, NEUROD2, BRAF, MCOLN1
	GO:0031295~T cell costimulation	EFNB2, EFNB1, ICOS
Cellular Compartment	GO:0005886~plasma membrane	DGKG, MRGPRF, NCMAP, IL27RA, GPR173, GJA3, BSG, MS4A12, CMKLR1, ENTPD2, CXADR, IL1R2, SYTL1, SLC9B2, MAG, AGTR1, APLNR, KCNE1, TSG101, TTYH3, CD151, GYPB, SLC22A1, FPR1, LPAR1, LYPD3, CD99L2, ADRB2, CBARP, EFNB2, SLCO1B1, EFNB1, EPB41L2, EPB41L3, ICOS, TMEM184A, SYT5, HSPA8, MPIG6B, AMBP, CAV2, MCAM, FZD7, SURF4, CYBRD1, TNFRSF10A, BRAF, TREML1, LRFN4, FXYD2, SSPN, CD200, SCN2B, SLC44A5, PTH1R, SLC2A5, SLC6A1, LITAF, SLC2A6, CX3CL1, SPN, HAVCR1, ERMAP, C11ORF24, FGB, OPALIN, MMP2, SLC2A12, GPBAR1, PTH2R, BTN3A3, BTN3A2, KCNAB3, SLC7A8, ADGRF5, NUMB, GPRC5D, PTGER4, PLPPR1, STAU2, PACC1, CXCR6, ACVR1B, RNF5, SLITRK3, PDPN, S1PR3, CACNG1, CACNG2, RELT, LAIR1, JAM2, SEMA4D, KCNIP1, KCNIP2, KIR3DL2, MCOLN1, CD4, FCGR2A, PIK3IP1, CD6, CD7, RAB9A, PLP2, FCGR2B, NECTIN4, CRB3
	GO:0016021~integral component of membrane	ZNF451, DPY19L2P2, MRGPRF, IL27RA, GPR173, SYNPR, GJA3, BSG, MS4A12, VSIG8, CMKLR1, STARD3, ENTPD2, IL1R2, SLC9B2, MAG, AGTR1, APLNR, KCNE1, RTN1, CD151, KCNE4, GYPB, SLC22A1, FPR1, LPAR1, LYPD3, SLC38A11, CD99L2, ADRB2, CBARP, SLCO1B1, EPB41L3, ICOS, C16ORF54, TMEM184A, SYT5, MPIG6B, CAV2, RHBDD1, TREX1, MCAM, FZD7, SURF4, CYBRD1, TNFRSF10A, DNAJC14, TREML1, LRFN4, FAM163A, CD200, SLC44A5, PTH1R, SLC2A5, SLC6A1, LITAF, SLC2A6, CX3CL1, SPN, TMEM108, HAVCR1, ERMAP, C11ORF24, OPALIN, SLC2A12, GPBAR1, PTH2R, BTN3A3, C1ORF162, BTN3A2, SYP, SLC7A8, ADGRF5, RFT1, GPRC5D, PTGER4, PLPPR1, SLC43A3, PACC1, TMEM72, CXCR6, RNF5, PDPN, RELT, LAIR1, JAM2, RPRM, CMTM4, TMEM176B, KIR3DL2, MCOLN1, TMEM154, CD4, FCGR2A, PIK3IP1, CD6, LPCAT4, CD7, FAM171B, PLP2, FCGR2B, NECTIN4, CRB3
	GO:0005887~integral component of plasma membrane	CD151, PLPPR1, PACC1, GYPB, SLC22A1, LPAR1, ADRB2, CXCR6, PTH1R, SLC6A1, ACVR1B, SLC2A5, NCMAP, IL27RA, EFNB2, SPN, EFNB1, SLCO1B1, GJA3, PDPN, BSG, S1PR3, CACNG1, JAM2, CMKLR1, CXADR, SEMA4D, CAV2, PTH2R, KIR3DL2, MCOLN1, MAG, CD4, FCGR2A, SLC7A8, CD6, SSPN, AGTR1, APLNR, FCGR2B, CD200
	GO:0009986~cell surface	FGB, KCNE1, CD151, AMBP, PACC1, LPAR1, CD99L2, TNFRSF10A, SLC6A1, ACVR1B, CX3CL1, SPN, TREML1, EFNB1, LRFN4, CD6, APOH, ADGRF5, SLITRK3, CACNG2, HAVCR1, CD200, JAM2
	GO:0016020~membrane	LC44A5, DGKG, PTH1R, SLC6A1, SLC2A5, SLC2A6, CX3CL1, SPN, SYNPR, BSG, H4C1, VSIG8, CMKLR1, H3C8, ENTPD2, IL1R2, SLC2A12, BTN3A3, SYP, C1ORF162, BTN3A2, DNAJC7, ADGRF5, AGTR1, VDAC3, FTL, PTGER4, CD151, STAU2, SLC22A1, TMEM72, FPR1, SLC38A11, ADRB2, ACVR1B, EFNB2, SLCO1B1, PDPN, RPRM, C16ORF54, HSPA8, CMTM4, CAV2, FZD7, CYBRD1, DNAJC14, MCOLN1, TMEM154, TREML1, CD4, CD6, LPCAT4, FAM163A, AGO2, CD7, FAM171B, PLP2, CD200
	GO:0016323~basolateral plasma membrane	SLCO1B1, CXADR, SLC7A8, SLC43A3, SLC22A1, PDPN, BSG, NUMB, PTH1R, SLC9B2
	GO:0043005~neuron projection	SYNPR, CXADR, PLPPR1, RTN1, STAU2, SMN2, BRAF, SYP, SLC6A1, MARK4, CX3CL1, CD200
	GO:0045121~membrane raft	EFNB1, MAG, CD4, KCNE1, CXADR, CAV2, PDPN, BSG, TNFRSF10A
	GO:0042383~sarcolemma	STAC, CAV2, BSG, SSPN, CACNG1, SLC2A5
	GO:0016324~apical plasma membrane	KCNE1, SLC7A8, KCNE4, SLC22A1, PDPN, CYBRD1, PTH1R, ADRB2, SLC2A5, CRB3, SLC9B2
	GO:0033270~paranode region of axon	MAG, EPB41L3, NCMAP
	GO:0036477~somatodendritic compartment	TMEM108, CACNG2, JAM2
	GO:0009897~external side of plasma membrane	FGB, SPN, CD4, CD6, MCAM, BTN3A3, CXCR6, BTN3A2, FCGR2B, ERMAP, IL27RA
	GO:0070062~extracellular exosome	TSG101, TTYH3, SLC2A5, CST4, SPN, RAB43, EFNB1, HPX, CYSRT1, APOH, EPB41L2, BSG, H4C1, CAMP, FGB, H3C8, HSPA8, ENTPD2, AMBP, SYTL1, CYBRD1, DNAJC7, FXYD2, AGO2, VDAC3, RAB9A, ALDOB, NECTIN4, GPRC5D, CRB3, FTL
	GO:0043235~receptor complex	GPBAR1, PTH1R, ADRB2, GPRC5D, MCOLN1, ACVR1B, IL27RA
	GO:0005925~focal adhesion	EFNB2, HSPA8, TLE2, CD151, CAV2, MCAM, EPB41L2, BSG, NUMB, CD99L2
	GO:0030672~synaptic vesicle membrane	SYT5, SYNPR, SYP, CBARP, SLC9B2
	GO:0044297~cell body	CXADR, BRAF, FCGR2B, CD200
	GO:0031528~microvillus membrane	SLC7A8, PDPN, SYTL1
	GO:0008076~voltage-gated potassium channel complex	KCNE1, KCNIP1, KCNIP2, KCNAB3
	GO:0098982~GABA-ergic synapse	LRFN4, SLITRK3, LPAR1, SLC6A1
	GO:0072562~blood microparticle	FGB, HSPA8, HPX, PRSS1, AMBP
	GO:0030425~dendrite	EFNB2, HSPA8, SHARPIN, RTN1, KCNIP1, KCNIP2, AGO2, TMEM108, MARK4
Molecular Function	GO:0005515~protein binding	ZNF451, IL27RA, RAB43, SYNPR, CYSRT1, RUSC1, BSG, BANF1, MS4A12, H4C1, PDK1, CMKLR1, STARD3, TLE2, ENTPD2, CXADR, IL1R2, SYTL1, MAT1A, KCTD4, SLC9B2, NPAS4, AGTR1, APLNR, VDAC3, FTL, NAA60, ZNF792, KCNE1, TSG101, RTN1, CD151, KCNE4, GYPB, CUL2, SLC22A1, FPR1, LPAR1, SLC38A11, CD99L2, ADRB2, EFNB2, EFNB1, HPX, SHARPIN, APOH, EPB41L2, EPB41L3, SMN2, DMAP1, ICOS, MARK4, C16ORF54, HSPA8, NIBAN3, MPIG6B, AMBP, RBPMS, CAV2, STAC, RHBDD1, FZD7, SURF4, CYBRD1, TNFRSF10A, BRAF, TREML1, KIF18A, LRFN4, FAM163A, AGO2, CIBAR1, CIBAR2, CD200, SLC44A5, PTH1R, SLC2A5, SLC6A1, LITAF, SLC2A6, CX3CL1, SPN, TMEM108, HAVCR1, ERMAP, C11ORF24, TGIF1, FGB, H3C8, STRBP, MMP2, PTH2R, OLIG1, BTN3A2, SYP, KCNAB3, DNAJC7, SLC7A8, GFOD1, ADGRF5, RFT1, NUMB, ALDOB, GPRC5D, PTGER4, PLPPR1, SLC43A3, STAU2, PACC1, TMEM72, RCHY1, ACVR1B, RNF5, CST4, SLITRK3, PDPN, HS1BP3, S1PR3, CACNG1, CACNG2, RELT, LAIR1, JAM2, RPRM, KRTAP10-5, SEMA4D, KCNIP1, CMTM4, KCNIP2, TMEM176B, CIMAP1A, KIR3DL2, MCOLN1, DHRS2, TMEM154, CD4, FCGR2A, PIK3IP1, CD6, CD7, CCDC170, RAB9A, PLP2, FCGR2B, NECTIN4, CRB3
	GO:0015459~potassium channel regulator activity	KCNE1, KCNIP1, KCNIP2, KCNE4, ADRB2, KCNAB3
	GO:0001618~virus receptor activity	EFNB2, CD4, CXADR, BSG, NECTIN4, HAVCR1
	GO:0044325~ion channel binding	KCNE1, KCNIP1, STAC, KCNIP2, KCNE4, KCNAB3, CBARP
	GO:0022857~transmembrane transporter activity	SLC44A5, SLCO1B1, SLC7A8, SLC43A3, SLC22A1, SLC2A5, SLC2A6
	GO:1902282~voltage-gated potassium channel activity involved in ventricular cardiac muscle cell action potential repolarization	KCNE1, KCNE4, SCN2B
	GO:0005355~glucose transmembrane transporter activity	SLC2A12, SLC2A5, SLC2A6
	GO:0004888~transmembrane signaling receptor activity	SPN, TREML1, CD4, FCGR2A, SEMA4D, FCGR2B, IL27RA
	GO:0042802~identical protein binding	ZNF792, SLC22A1, ADRB2, SLC6A1, RNF5, LITAF, SHARPIN, CYSRT1, APOH, SMN2, BANF1, KRTAP10-5, CXADR, SEMA4D, KCNIP2, CYBRD1, TNFRSF10A, BRAF, SYP, MAT1A, MCOLN1, SLC9B2, CD4, CD6, ALDOB, NECTIN4, FTL
	GO:0005102~receptor binding	FGB, MAG, CXADR, SEMA4D, PIK3IP1, PDPN, BTN3A3, BTN3A2, ERMAP, CX3CL1
	GO:0004991~parathyroid hormone receptor activity	PTH2R, PTH1R
	GO:0038023~signaling receptor activity	CD4, SEMA4D, BSG, CD7, APLNR, TNFRSF10A, CMKLR1
	GO:0044877~macromolecular complex binding	NPAS4, KCNE1, TSG101, SHARPIN, KCNIP2, CUL2, ADRB2, FCGR2B, RNF5
	GO:0004930~G-protein coupled receptor activity	FZD7, LPAR1, FPR1, MRGPRF, PTH2R, CXCR6, ADRB2, GPR173, ADGRF5, AGTR1, APLNR, S1PR3, GPRC5D, CMKLR1
	GO:0008289~lipid binding	DGKG, STARD3, CD4, APOH, S1PR3, MCOLN1
	GO:0042803~protein homodimerization activity	STARD3, NAA60, TSG101, AMBP, RBPMS, CAV2, TREX1, PTH1R, ADRB2, RCHY1, MAG, CD4, BANF1

**Table A4:** GO analysis of hits identified during MAPPIT and KISS screens against the HEV ORF4 protein.

Annotation Category	GO Term	Gene Name
Biological Process	GO:0000381~regulation of alternative mRNA splicing, via spliceosome	RBFOX1, RBFOX2, CELF4, CELF5
	GO:0045862~positive regulation of Proteolysis	FBXW11, STUB1, BTRC
	GO:0045892~negative regulation of transcription, DNA-templeted	LBH, RBFOX2, HEY1, FBXW11, EPC1, BTRC, WTIP, GATAD2A
	GO:0009615~response to virus	RPS15A, PRKRA, ADAR, CCT5
	GO:0043161~proteasome-mediated ubiquitin-dependent protein catabolic process	SHARPIN, FBXW11, STUB1, BTRC, KLHL20
	GO:0006397~mRNA processing	RBFOX1, RBFOX2, CELF4, SMN2, ADAR
	GO:0016567~protein ubiquitination	FBXW11, MKRN3, STUB1, BTRC, LZTR1, RCHY1, KLHL20
	GO:0007010~cytoskeleten organization	DES, ZMYM6, WTIP, KLHL20
	GO:0016236~macroautophagy	TSG101, CHMP4C, ZFYVE1
	GO:0000209~protein polyubiquitination	FBXW11, MKRN3, STUB1, BTRC
	GO:0042753~positive regulation of circadian rhythm	FBXW11, BTRC
	GO:0035455~response to interferon-alpha	ADAR, KLHL20
	GO:0000122~negative regulation of transcription from RNA polymerase II promotor	TSG101, HEY1, DDX20, ZBTB42, EPC1, ZNF3, MEIS2, GATAD2A, THAP11
Cellular Compartment	GO:0005737~cytoplasm	ITSN2, HSPB6, CELF4, CELF5, MSI1, ADAR, MSI2, PPP1CC, LBH, HEY1, EPC1, METTL16, LZTS2, BTRC, CEP55, SH3GLB2, RBFOX1, RBFOX2, DYNLT3, TRAF1, PRKAR1B, PSME1, BLZF1, TSG101, FBLIM1, CAMK2A, DDX20, C10ORF88, DAZ3, ZBTB42, RCHY1, KLC1, SAV1, RPS15A, GNA11, SMN2, LPXN, CTAG1B, PDLIM5, ZC3H14, PIBF1, CCT5, CARD9, KLHL20, DES, IMPDH1, PRKRA, STUB1, CIBAR1
	GO:0005829~cytosol	ITSN2, TSG101, HSPB6, FBLIM1, CAMK2A, DDX20, MSI2, RCHY1, KLC1, SAV1, KRT83, PPP1CC, RPS15A, SHARPIN, SMN2, CEP72, PLXNA1, LPXN, LZTS2, BTRC, PDLIM5, CCT5, SH3GLB2, RBFOX2, RBPMS, FBXW11, KRTAP10-9, CARD9, TRAF1, KLHL20, THAP11, DES, PRKAR1B, DDAH2, IMPDH1, PRKRA, CHMP4C, PSME1, STUB1
	GO:0005634~nucleus	HSPB6, CAMK2A, NAB2, DDX20, CELF4, CELF5, DAZ3, ZBTB42, MSI1, ADAR, ZNF3, RCHY1, SAV1, PPP1CC, LBH, HEY1, ZMYM6, SMN2, EPC1, LPXN, METTL16, BTRC, ZC3H14, PIBF1, RBFOX1, RBFOX2, FBXW11, DYNLT3, OLIG1, WTIP, MEIS2, GATAD2A, DES, IMPDH1, PRKRA, STUB1, CIBAR1, BLZF1
	GO:0090543~Flemming body	TSG101, CHMP4C, CEP55
	GO:0000151~ubiquitin ligase complex	SHARPIN, FBXW11, STUB1, RCHY1
	GO:0005771~multivesicular body	TSG101, PRKAR1B, CHMP4C
	GO:0030018~Z-disc	DES, SMN2, STUB1, PDLIM5
	GO:0005813~centrosome	ITSN2, FBXW11, CEP72, LZTS2, PIBF1, CCT5, CEP55
	GO:0000776~kinetochore	PPP1CC, FBXW11, DYNLT3, CHMP4C
	GO:0005654~nucleoplasm	SH3GLB2, RBFOX2, RBPMS, CAMK2A, NAB2, DDX20, CELF4, ZBTB42, ADAR, RCHY1, GATAD2A, THAP11, PPP1CC, RPS15A, HEY1, PRKRA, SMN2, PSME1, CEP72, PLXNA1, EPC1, STUB1, BTRC, BLZF1
	GO:1990904~ribonucleoprotein complex	CELF4, MKRN3, CELF5, ZC3H14
	GO:0030496~midbody	PPP1CC, CHMP4C, LZTS2, CEP55
	GO:0032797~SMN complex	DDX20, SMN2
	GO:0097504~Gemini of coiled bodies	DDX20, SMN2
Molecular Function	GO:0005515~protein binding	ITSN2, NAB2, IHH, CELF5, MSI1, ADAR, MSI2, ZFYVE1, PPP1CC, LBH, CYSRT1, HEY1, EPC1, LZTS2, BTRC, SMCO1, CEP55, LINC02875, SH3GLB2, RBFOX1, RBFOX2, FBXW11, DYNLT3, OLIG1, TRAF1, THAP8, PRKAR1B, DDAH2, PPP1R3B, CHMP4C, PSME1, PRR23E, NUP54, METTL27, BLZF1, TSG101, FBLIM1, CAMK2A, MOSPD2, DDX20, C10ORF88, DAZ3, ZBTB42, ZNF3, RCHY1, KLC1, KRT83, SAV1, FAM168A, RPS15A, SHARPIN, GNA11, MGAT5B, SMN2, CEP72, LPXN, CTAG1B, PDLIM5, ZC3H14, PIBF1, CCT5, RBPMS, KRTAP10-9, MIIP, CARD9, WTIP, MEIS2, GATAD2A, KLHL20, THAP11, DES, PRKRA, CCDC170, CORO6, MKRN3, NMUR2, STUB1, LZTR1, CIBAR1
	GO:0042802~identical protein binding	SH3GLB2, DYNLT3, CARD9, CAMK2A, NAB2, C10ORF88, MSI1, TRAF1, MSI2, ZNF3, SAV1, SHARPIN, DES, CYSRT1, IMPDH1, PRKRA, SMN2, MKRN3, NUP54, CTAG1B, PIBF1, CEP55
	GO:0003723~RNA binding	RBFOX1, RBFOX2, RBPMS, DDX20, CELF4, CELF5, ALG13, ADAR, MSI1, MSI2, PPP1CC, RPS15A, IMPDH1, PRKRA, SMN2, MKRN3, METTL16, ZC3H14
	GO:0003729~mRNA binding	RBFOX1, RBFOX2, RBPMS, CELF4, CELF5, MSI1, MSI2
	GO:0004842~ubiquitin-protein transferase activity	SHARPIN, FBXW11, STUB1, TRAF1, BTRC, RCHY1, KLHL20
	GO:0042803~protein homodimerization activity	TSG101, RBPMS, HSPB6, PRKRA, CARD9, CAMK2A, CHMP4C, STUB1, RCHY1
	GO:0046983~protein dimerization activity	HEY1, FBXW11, OLIG1, BTRC

**Table A5:** Functional annotation clustering of the HEV ORF candidate interactors.

**Annotation cluster 1**	**Enrichment score: 10.14**	**Amount of hits**
GOTERM_CC_DIRECT	plasma membrane	138
UP_KW_CELLULAR_COMPONENT	cell membrane	103
UP_KW_CELLULAR_COMPONENT	Membrane	170
**Annotation cluster 2**	**Enrichment score: 2.38**	**Amount of hits**
GOTERM_BP_DIRECT	viral entry into host cell	7
UP_KW_MOLECULAR_FUNCTION	host cell receptor for virus entry	6
GOTERM_MF_DIRECT	virus receptor activity	6
**Annotation cluster 3**	**Enrichment score: 1.9**	**Amount of hits**
GOTERM_BP_DIRECT	miRNA loading onto RISC involved in gene silencing by miRNA	3
UP_KW_BIOLOGICAL_PROCESS	RNA-mediated gene silencing	5
GOTERM_BP_DIRECT	pre-miRNA processing	3
GOTERM_MF_DIRECT	double-stranded RNA binding	5
**Annotation cluster 4**	**Enrichment score: 1.72**	**Amount of hits**
GOTERM_MF_DIRECT	mRNA binding	11
UP_KW_MOLECULAR_FUNCTION	RNA-binding	21
GOTERM_MF_DIRECT	RNA binding	27
**Annotation cluster 5**	**Enrichment score: 1.7**	**Amount of hits**
UP_KW_MOLECULAR_FUNCTION	voltage-gated channel	8
GOTERM_BP_DIRECT	membrane repolarization during ventricular cardiac muscle cell action potential	3
GOTERM_MF_DIRECT	voltage-gated potassium channel activity involved in ventricular cardiac muscle cell action potential repolarization	3
GOTERM_BP_DIRECT	regulation of heart rate by cardiac conduction	3
**Annotation cluster 6**	**Enrichment score: 1.62**	**Amount of hits**
GOTERM_MF_DIRECT	potassium channel regulator activity	6
GOTERM_BP_DIRECT	regulation of potassium ion transmembrane transport	4
GOTERM_MF_DIRECT	ion channel binding	8
UP_KW_MOLECULAR_FUNCTION	voltage-gated channel	8
GOTERM_BP_DIRECT	potassium ion export across plasma membrane	3
UP_KW_BIOLOGICAL_PROCESS	Ion transport	17
UP_KW_BIOLOGICAL_PROCESS	Potassium transport	6
UP_KW_MOLECULAR_FUNCTION	Ion channel	11
UP_KW_MOLECULAR_FUNCTION	Potassium channel	4
GOTERM_CC_DIRECT	voltage-gated potassium channel complex	4
GOTERM_MF_DIRECT	voltage-gated potassium channel activity	3
GOTERM_BP_DIRECT	potassium ion transmembrane transport	3
**Annotation cluster 7**	**Enrichment score: 1.53**	**Amount of hits**
UP_KW_MOLECULAR_FUNCTION	receptor	43
GOTERM_BP_DIRECT	positive regulation of cytosolic calcium ion concentration	8
UP_KW_MOLECULAR_FUNCTION	G-protein coupled receptor	19
UP_KW_MOLECULAR_FUNCTION	transducer	20
GOTERM_BP_DIRECT	G-protein coupled receptor signaling pathway	20
GOTERM_MF_DIRECT	G-protein coupled receptor activity	16
**Annotation cluster 8**	**Enrichment score: 1.5**	**Amount of hits**
GOTERM_MF_DIRECT	glucose transmembrane transporter activity	3
UP_KW_BIOLOGICAL_PROCESS	sugar transport	4
GOTERM_BP_DIRECT	glucose transmembrane transport	3
**Annotation cluster 9**	**Enrichment score: 1.04**	**Amount of hits**
GOTERM_BP_DIRECT	chemotaxis	7
GOTERM_BP_DIRECT	chemokine-mediated signaling pathway	3
GOTERM_BP_DIRECT	inflammatory response	8
**Annotation cluster 10**	**Enrichment score: 0.96**	**Amount of hits**
UP_KW_MOLECULAR_FUNCTION	developmental protein	21
UP_KW_MOLECULAR_FUNCTION	developmental protein	21
UP_KW_BIOLOGICAL_PROCESS	differentiation	16
**Annotation cluster 11**	**Enrichment score: 0.94**	**Amount of hits**
GOTERM_BP_DIRECT	positive regulation of proteolysis	3
GOTERM_BP_DIRECT	post-translational protein modification	4
GOTERM_BP_DIRECT	SCF-dep. proteasomal ubiquitin-dep. protein catabolic process	4
GOTERM_MF_DIRECT	ubiquitin-protein transferase activity	8
GOTERM_BP_DIRECT	protein polyubiquitination	6
GOTERM_BP_DIRECT	proteasome-mediated ubiquitin-dep. protein catabolic process	6
GOTERM_MF_DIRECT	ubiquitin protein ligase activity	7
**Annotation cluster 12**	**Enrichment score: 0.79**	**Amount of hits**
GOTERM_MF_DIRECT	protein binding involved in protein folding	4
GOTERM_BP_DIRECT	protein refolding	3
UP_KW_MOLECULAR_FUNCTION	chaperone	7
GOTERM_MF_DIRECT	unfolded protein binding	5
GOTERM_BP_DIRECT	protein folding	5
UP_KW_BIOLOGICAL_PROCESS	stress response	3
GOTERM_MF_DIRECT	ATPase activity	6
**Annotation cluster 13**	**Enrichment score: 0.68**	**Amount of hits**
GOTERM_CC_DIRECT	ubiquitin ligase complex	5
GOTERM_MF_DIRECT	ubiquitin-protein transferase activity	8
GOTERM_BP_DIRECT	protein destabilization	3
GOTERM_BP_DIRECT	proteasome-mediated ubiquitin-dep. protein catabolic process	6
GOTERM_BP_DIRECT	protein ubiquitination	10
UP_KW_BIOLOGICAL_PROCESS	Ubl conjugation pathway	13
GOTERM_MF_DIRECT	ubiquitin protein ligase activity	7
GOTERM_BP_DIRECT	ubiquitin-dependent protein catabolic process	5
**Annotation cluster 14**	**Enrichment score: 0.62**	**Amount of hits**
GOTERM_BP_DIRECT	cell projection organization	4
GOTERM_CC_DIRECT	ciliary basal body	5
UP_KW_BIOLOGICAL_PROCESS	cilium biogenesis/degradation	5
GOTERM_CC_DIRECT	centriole	4
GOTERM_BP_DIRECT	cilium assembly	4
**Annotation cluster 15**	**Enrichment score: 0.59**	**Amount of hits**
GOTERM_BP_DIRECT	regulation of alternative mRNA splicing, via spliceosome	4
GOTERM_BP_DIRECT	mRNA processing	6
UP_KW_BIOLOGICAL_PROCESS	mRNA processing	8
UP_KW_BIOLOGICAL_PROCESS	mRNA splicing	6
GOTERM_BP_DIRECT	RNA splicing	4
**Annotation cluster 16**	**Enrichment score: 0.59**	**Amount of hits**
UP_KW_BIOLOGICAL_PROCESS	differentiation	16
UP_KW_BIOLOGICAL_PROCESS	spermatogenesis	6
GOTERM_BP_DIRECT	cell differentiation	9
GOTERM_BP_DIRECT	spermatogenesis	6
**Annotation cluster 17**	**Enrichment score: 0.34**	**Amount of hits**
GOTERM_BP_DIRECT	microtubule-based movement	3
UP_KW_CELLULAR_COMPONENT	microtubule	6
UP_KW_MOLECULAR_FUNCTION	motor protein	3
**Annotation cluster 18**	**Enrichment score: 0.26**	**Amount of hits**
GOTERM_CC_DIRECT	midbody	5
UP_KW_BIOLOGICAL_PROCESS	cell division	7
GOTERM_BP_DIRECT	cell cycle	6
UP_KW_BIOLOGICAL_PROCESS	mitosis	5
GOTERM_BP_DIRECT	cell division	6
UP_KW_BIOLOGICAL_PROCESS	cell cycle	8
**Annotation cluster 19**	**Enrichment score: 0.21**	**Amount of hits**
UP_KW_BIOLOGICAL_PROCESS	adaptive immunity	9
UP_KW_BIOLOGICAL_PROCESS	immunity	13
GOTERM_BP_DIRECT	adaptive immune response	6
**Annotation cluster 20**	**Enrichment score: 0.08**	**Amount of hits**
GOTERM_MF_DIRECT	kinase activity	4
GOTERM_BP_DIRECT	protein phosphorylation	7
UP_KW_MOLECULAR_FUNCTION	kinase	9
UP_KW_MOLECULAR_FUNCTION	serine/threonine-protein kinase	4
GOTERM_MF_DIRECT	protein serine/threonine kinase activity	4
**Annotation cluster 21**	**Enrichment score: 0.08**	**Amount of hits**
GOTERM_CC_DIRECT	extracellular region	28
GOTERM_CC_DIRECT	extracellular space	25
UP_KW_CELLULAR_COMPONENT	secreted	23
**Annotation cluster 22**	**Enrichment score: 0.05**	**Amount of hits**
UP_KW_MOLECULAR_FUNCTION	repressor	11
GOTERM_BP_DIRECT	positive regulation of transcription from RNA polymerase II promoter	15
GOTERM_BP_DIRECT	regulation of transcription, DNA-templated	12
GOTERM_MF_DIRECT	transcriptional activator activity, RNA polymerase II transcription regulatory region sequence-specific binding	6
GOTERM_CC_DIRECT	chromatin	10
UP_KW_BIOLOGICAL_PROCESS	transcription regulation	26
UP_KW_MOLECULAR_FUNCTION	DNA-binding	22
UP_KW_BIOLOGICAL_PROCESS	transcription	26
GOTERM_MF_DIRECT	RNA polymerase II transcription factor activity, sequence-specific DNA binding	12
GOTERM_MF_DIRECT	RNA polymerase II core promoter proximal region sequence-specific DNA binding	11
GOTERM_MF_DIRECT	sequence-specific double-stranded DNA binding	4
GOTERM_MF_DIRECT	transcription factor activity, sequence-specific DNA binding	4
GOTERM_BP_DIRECT	regulation of transcription from RNA polymerase II promoter	13

**Table A6:** Described functions in viral infection of HEV ORF candidate interactors.

Gene Symbol	HEV Interacting Protein	Function in Viral Infection	Reference
UBR2	ORF2	- Human immunodeficiency virus 1 (HIV-1) infection was decreased in UBR2-depleted cells	[68]
FTL	ORF2, ORF3	- Interacts with several proteins and domains of HEV (ORF1, macro, protease, helicase, ORF3). It was proposed that macro domain prevents ferritin from entering into circulation and helping attenuating host immune response - In vitro overexpression of FTL can enhance Hepatitis B virus (HBV) attachment	[26,28,69,70]
TNFRSF10B	ORF2	- Expression reduced in liver tissue from chronic HBV and HBV infected cell lines - HBV X protein degrades TNFRSF10B via macroautophagy-mediated degradation, allowing survival of virus-infected cells - Hantavirus inhibits TRAIL-mediated killing of infected cells by downregulating TNFRSF10B - HBV core protein inhibits TRAIL-induced apoptosis of hepatocytes by blocking TNFRSF10B expression - Hepatitis C virus (HCV) upregulates TNFRSF10B, sensitizes host cells to TRAIL-induced apoptosis	[71,72,73]
TOMM34	ORF2	- Upregulated in HCV-induced hepatocellular carcinoma (HCC) - Upregulated in HIV-infected CD4 T-cells - Increased phosphorylation in Dengue Virus (DENV)-2 infected cells	[74,75,76]
TIGIT	ORF2	- Functionally linked to IL-10 expression, known to promote virus persistence in vitro - TIGIT modulation alters phenotype and cytokine profile of T cells during Influenza and chronic Lymphocytic choriomeningitis virus (LCMV) - Regulates NK cell function in chronic HBV infection - Blocking TIGIT enhances NK cell activity against HIV-infected T cells	[77,78,79]
FCGR2A	ORF2, ORF3	- Variants are associated with thrombocytopenia in DENV - Variants are associated with severe Respiratory syncytial virus (RSV) in Argentina - Enhanced FCGR2A signaling in HIV viremic controllers	[80,81]
ANXA4	ORF2	- Downregulated in human monocytic cells infected with DENV - Upregulated in a proteomic study analyzing saliva from COVID-19 patients - Higher levels of serum ANXA4 in HCV-related HCC	[82,83,84]
CMTM6	ORF2	- Involved in the PLD1 upregulation after entecavir treatment during HBV infection, may be a mechanism of hepatocytes to subvert immune surveillance	[85]
TTC1	ORF2	- Upregulated in A549 cells after Influenza virus	[86]
CAPNS2	ORF2	- Downregulated Human papillomavirus 16 (HPV16) infection - Upregulated during Human cytomegalovirus (HCMV) infection	[87,88]
PSMA5	ORF2	- Recognizes oxidized ApoB (together with PSMA6) that is induced upon HCV infection, which is known to enhance lipid accumulation and promote viral production - Increased in PBMCs from COVID-19	[89,90]
FUBP3	ORF2	- Knockdown leads to decreased Japanese encephalitis virus (JEV), co-localizes with viral proteins and assists in viral replication complex - Host restriction factor in Porcine epidemic diarrhea virus (PEDV) infection, degrades PEDV-encoded nucleocapsid via E3 ubiquitin ligase recruitment, and regulates IFN-1 signaling pathway by interacting with TNF-TRAF3 expression - Interacts with enterovirus EV71 5′UTR and knockdown and leads to decreased EV71 IRES activity; might therefore serve as IRES regulator	[91,92,93]
LGALS8	ORF2	- Controls autophagy during adenovirus cell entry through viral capsid. PPXY motif in this protein is critical for efficient evasion of autophagic sequestration. Depletion of LGALS8 rescues infectivity of a PPXY-mutant virus	[94]
STUB1	ORF2, ORF4	- Targeted by the SUMO-interacting motif of EBNA1 to maintain Epstein–Barr Virus (EBV) latency - Bovine ephemeral fever (BEFV) triggers RACK1 expression, which upregulates STUB1, thereby promoting MAVS degradation and IFN type I inhibition - STAT4 can interact with STUB1 to block RIG-I in RNA virus infection	[95,96,97]
STAT5B	ORF2	- Reduction in STAT5B expression in HIV-infected patients - STAT5B activation promotes virus amplification of insect viruses, whereas suppression inhibits apoptosis and reduces virus accumulation, linked with JAK-STAT pathway - Target of SARS-CoV-2 encoded miRNAs	[98,99,100,101]
ADRB2	ORF3	- Upregulated by NK cells in early mouse CMV infection - Downregulates innate immune response and reduces host resistance to viral infection	[102,103]
PDPN	ORF3	- Incorporated into HIV released from HEK293T, required for efficient binding to attachment factor CLEC-2	[104]
IL27RA	ORF3	- IL-27 enables optimal antiviral immunity early and late after persistent virus infection	[105,106]
ICOS	ORF3	- Enhances all basic T cell responses - Antibody responses affected for LCMV, vesicular stomatitis virus (VSV) and influenza in ICOS-knockout mice	[107]
SLITRK3	ORF3	- Downregulated in cerebrospinal fluid in patients with varicella zoster virus (VZV) meningitis - Downregulated in HCV+ liver tissues (cirrhotic state) compared to normal adjacent tissues	[108,109]
FCGR2B	ORF3	- Upregulated upon HBsAg-anti-HBs complex treatment in dendritic cells from an HBsAg transgenic mouse model and peripheral B cells from patients with chronic hepatitis B - Upregulation in patients with mild COVID-19 compared to asymptomatic cases - Simian virus vaccine-induced Abs form virus-specific immune complexes upon reaction with challenge virus and interact with FCGR2B to downregulate innate immune and inflammatory responses	[110,111,112]
CD7	ORF3	- Downregulation of CD7 in combination with activated CD8+ T-cell population might be attributed to acute EBV infection - Important for the HIV-1 fusion process that results in syncytium formation - HTLV-I results in loss of CD7 expression, further inducing Akt/Bad phosphorylation, which generates resistance to apoptosis - Upregulated upon IBDV infection	[113,114,115,116]
SLC9B2	ORF3	- Upregulated in cells treated with SARS-CoV-2 nucleocapsid protein	[117]
LPAR1	ORF3	- Ligand (LPA) concentrations are upregulated upon HCV or SARS-CoV-2, binding of LPA to LPAR1 restricts type I and type III IFN.	[118]
RBPMS	ORF3, ORF4	- Identified as a host stimulatory factor for West Nile Virus (WNV) by RNAi screen. - Interacts with EBV proteins EBNA3B, BALF4 and BDLF4 and acts as latent–lytic switch in EBV by negatively interacting with STAT3	[119,120]
CMKLR1	ORF3	- Co-receptor for several SIV strains and HIV-1 strain - Lower levels of CMKLR1 in HBV HCC tissues	[121,122]
APLNR	ORF3	- Alternative coreceptor with CD4 for HIV-1	[123]
SLCO1B1	ORF3	- Significant association with survival in HBV-mediated HCC	[124]
PRRS1	ORF3	- Related to genetic variant and pancreatitis in HEV-infected individual - Protein associated with the Pseudorabies virion	[125,126]
EPB41L3	ORF3	- Associated with asthma in RSV latent infection - Differentially expressed in blood of older individuals that might predispose to severe cases of COVID-19 with vascular and immune alterations	[127,128]
CACNG1	ORF3	- Expression levels downregulated in SH-SY5Y cells infected with VZV	[129]
DNAJC14	ORF3	- Pivotal cellular cofactor for replication and maintenance of pestiviruses, interacts with viral protein NS2 - Interacts with members of the Flaviviridae family and inhibits viral replication	[130,131]
SLC2A5	ORF3	- Differentially expressed after SARS-CoV-2 infection	[132]
RTN1	ORF3	- Previous interaction with HEV ORF1 and RdRp - Identified as a regulator of HCV propagation	[133]
STARD3	ORF3	- Host dependency factor for HCV, affects entry and replication	[134]
CXCR6	ORF3	- Co-receptor for HIV-1 and HIV-2 - Influences inflammatory response in context of influenza virus - NK cell memory of viruses depends on this receptor - Effector T cells in the liver express more CXCR6	[135,136,137,138]
CST4	ORF3	- Upregulated in nasal aspirates from influenza A virus (IAV)-infected children	[139]
FPR1	ORF3	- Upregulated after mono-HIV and HCV infection - Higher expression in HTLV-1 patients - Differentially expressed in bronchoalveolar lavage from severe-COVID-19 patients	[140,141,142]
TMEM176B	ORF3	- Protective host factor in coronavirus infection by controlling inflammasome activation	[143]
RNF5	ORF3	- V protein of Newcastle disease virus (NDV) or PB1 of IAV recruit RNF5 to polyubiquitinate and degrade MAVS, inhibiting type I IFN - Sendai Virus and pseudorabies virus recruit RNF5 to inhibit STING-mediated antiviral immunity	[66,67,144,145]
CXADR	ORF3	- Coxsackievirus and adenovirus receptor,	[44]
ZAR1L	ORF3	- Upregulated in ZIKV-infected JEG-3 cells.	[146]
PDK1	ORF3	- JEV infection of neurons results in upregulation of PDK1 abundance and promotes neuronal apoptosis, which contributes to JEV morbidity and mortality - mRNA of PDKs are elevated upon HCV infection and result in promoting serine and glycine synthesis, thereby contributing to a favorable environment for HCV replication	[147,148]
TGIF1	ORF3	- downregulation in HCV-infected patients in context of liver fibrosis - upregulated upon SARS-CoV-2 infection - Bovine viral diarrhea virus-infected cells have reduced TGIF1 expression, which might cause gastrulation defects and embryonic lethality	[149,150,151]
FZD7	ORF3	- Overexpressed in HCC tumors, mostly related to chronic hepatitis B virus infection	[152]
SHARPIN	ORF3, ORF4	- PRRSV NSP1 impairs interaction between HOIP and SHARPIN, thereby reducing LUBAC-dependent ubiquitination of NEMO and suppressing NF-kB activation - LUBAC inhibits virus-induced interferon in case of Sendai virus and VSV infection - LUBAC is essential for induction of interferon in case of human norovirus infection	[58,59,153,154]
TNFRSF10A	ORF3	- Identified as HIV-1 restriction factor in cell culture - Upregulation of TNFRSF10A (and related receptor and associated ligand) upon RSV infection; linked with apoptosis in early viral replicative cycle	[155,156,157]
SCN2B	ORF3	- Identified as druggable gene for viral hemorrhagic septicemia virus (VHSV) - Differentially expressed in HIV-infected individuals suffering from HIV-encephalitis and proposed as potential therapeutic target	[158,159]
OCT1	ORF3	- Downregulated protein abundance and mRNA levels in HCV-infected liver samples.	[160]
PLPPR1	ORF3	- Upregulated expression in cells expressing flavivirus capsid proteins	[161]
ADGRF5	ORF3	- Upregulated in glioblastoma cells susceptible to infection with a strain of measles virus	[162]
ENTPD2	ORF3	- Reduced levels in COVID-19 patients	[163]
SLC38A11	ORF3	- Differentially expressed in influenza-stimulated B-cells	[164]
SLC44A5	ORF3	- Interacts with RSV NS1, also identified with MAPPIT and KISS	[5]
EPB41L2	ORF3	- Enriched in proteomic study of HCV core + 1/ARFP protein, possibly related to cirrhosis - Identified in a proteomic screen as a cellular factor incorporated during Nipah virus budding	[165,166]
GPBAR1	ORF3	- Zebrafish orthologue involved in antiviral activity against spring viremia of carp virus (SVCV) - Downregulated expression in relation to SARS-CoV-2, but upregulated in influenza, SARS-CoV-1, RSV and rhinovirus - Identified as ISG with increased expression after viral infection. Deficiency results in increased VSV and Herpe simplex virus (HSV)-1 replication, probably by facilitating IFN production through Akt/IRF3	[167,168,169]
LITAF	ORF3	- Interacts with NS26 of grass carp reovirus (GCRV) - Alters the subcellular localization of frog virus 3 75L to late endosomes/lysosomes and co-localizes with LITAF. - Upregulation in chickens infected with highly pathogenic avian influenza virus, but downregulated in ducks (who show a milder infection compared to chickens)	[170,171,172]
APOM	ORF3	- Levels of serum APOM in patients with HBV are elevated compared to healthy individuals; APOM promotor activity, mRNA and protein expression simulated in HBV-transfected cells, and reduces expression of HBV S and E proteins and synthesis of viral DNA - APOM is associated with HCV particles, as identified by affinity purification–mass spectrometry, and is required for HCV production; interacts with HCV E2 protein	[173,174]
SYP	ORF3	- Borna disease virus (BDV) induces decrease in expression of SYP, followed by loss of cortical neurons - HSV-1-infected neurons have reduced SYP expression - Lower immunoreactivity of SYP in the cortex resulting from feline immunodeficiency virus co- and superinfection	[175,176,177]
NEUROD2	ORF3	- Downregulated in Zika virus (ZIKV)-infected brains having neurodevelopmental phenotypes	[178]
RCHY1	ORF3, ORF4	- Interacts with SARS-unique domain (SUD) and papain-like protease (PLpro), association increases RCHY1 stability and increases its ubiquitination function and degradation of p53 - Host factor restricting IAV infection by modulating ubiquitination of nucleoproteins - ORF3 protein of porcine circovirus type 2 competes with p53 in binding RCHY1 to deregulate p53 and apoptosis - Interacts with C-terminal domain of the measles virus phosphoprotein in Y2H, thereby stabilizing RCHY2 by preventing its ubiquitination	[179,180,181,182]
S1PR3	ORF3	- S1PR3 mRNA overexpressed in EBV-positive nasopharyngeal carcinoma (NPC) patient-derived xenografts and primary NPC tissues; knockdown suppresses AKT activation and Sphingosine-1-phosphate induced migration of NPC cells	[183]
ACVR1B	ORF3	- HCMV-induced miRNA (miRUL148D) targets ACVR1B, specifically inhibits ACVR1B expression in latently infected cells to limit pro-inflammatory cytokine production	[184]
EFNB1	ORF3	- Receptor for Cedar virus - Strongly upregulated after infectious pancreatic necrosis virus (IPNV) infection of Chinook salmon embryonic cells	[185]
DNAJC7	ORF3	- Fowl adenovirus serotype 4 (FAdV-4) PPI method shows interaction HSP70 and co-factor DNAJC7, which negatively regulate viral replication. Hexon protein interaction with HSP70 and assistance of DNAJC7 results in suppression of Hexon protein through the autophagy pathway - The cap protein of Porcine circovirus type 2 interacts with DNAJC7 and allows one to inhibit PKR activation	[186,187]
CAV2	ORF3	- Together with other caveolae-associated proteins incorporated into Tiger frog virus (TFV), they might be a restriction factor that affects viral release - Host factor of the RSV matrix protein; siRNA knockdown reduces RSV virus production - Identified as a protein, together with other proteins associated with lipid rafts, associated with HCV-induced autophagosomes (to enhance viral replication)	[188,189,190]
VDAC3	ORF3	- HBV X protein binds VDAC3 and alters mitochondrial membrane potential. It also activates transcription factors including STAT3 and NFkB - Interacts with EV71 2B protein, to enhance mitochondrial reactive oxygen species generation, which promotes viral replication.	[191,192]
SPN	ORF3	- IAV binds to human neutrophils (PMNs), mediated by glycoproteins including SPN - SPN-deficient CD4+ T-cells have an enhanced susceptibility to HIV	[193,194]
MAG	ORF3	- Binds HIV-1 gp120; may serve as receptor in peripheral nerve and be associated with neuropathy - Binds VZV glycoprotein to mediate entry of neurotropic herpesviruses	[195,196]
BSG	ORF3	- Involved in infection of measles and HCMV	[45,46]
BTN3A2	ORF3	- Polymorphisms of this gene may alter susceptibility to HCV infection	[197]
CRB3	ORF3	- CRB3 is part of complex (CRB3-PALS1-PATJ). The SARS-CoV-2 E protein competes with CRB3 for binding PALS1, which might play a role in disrupting lung epithelium in SARS patients	[198]
NUMB	ORF3	- ZIKV induces degradation of the NUMB protein, which might contribute to neurogenesis - The HBV e antigen and its precursors can interact with NUMB to impair stability of p53 and suppress its dependent apoptosis, thereby contributing to hepatocellular carcinoma	[199,200]
NECTIN4	ORF3	- Receptor for measles virus	[48]
TSG101	ORF3, ORF4	- Well known as interaction partner of ORF3 to mediate viral release	[15]
PTH1R	ORF3	- HTLV-1 infection upregulates PTH1R in lymphocytes, indicating a potential autocrine role for PTHrP	[201]
PTGER4	ORF3	- Receptor for PGE2, which is a modulator for viral infections	[202]
SURF4	ORF3	- involved in the replication of HCV by interacting with the HCV NS4B protein	[203]
CX3CL1	ORF3	- upregulated upon HEV infection in primary human hepatocytes - HIV Tat protein suppresses CX3CL1-CX3CR1 axis in microglia, which serve as viral reservoirs and producers of neurotoxins - Differentially expressed in HVB-infected hepatoma cells	[204,205,206]
CYBRD1	ORF3	- SNPs in CYBRD1 related with higher prevalence of HCC in patients with chronic HCV	[207]
HAVCR1	ORF3	- Well-known receptor for enveloped and non-enveloped viruses by binding phosphatidylserine, including HEV	[49,50]
AGTR1	ORF3	- During SARS coronavirus-2/SARS-CoV-2 infection, it is able to recognize and internalize the complex formed by secreted ACE2 and SARS-CoV-2 spike protein through DNM2/dynamin 2-dependent endocytosis	[47]
BANF1	ORF3	- Antiviral effector against vaccinia virus due to its DNA-binding activity, vaccina’s B1 kinase can inactivate BANF1 by phosphorylation - Interferes with HSV DNA replication - HBV inhibits BANF1, by downregulation of miR-203 by the X protein	[208,209,210]
BRAF	ORF3	- Cellular interactor of NS3 from bluetongue virus - Dysregulated expression in Merker cell polyomavirus positive samples from non-small cell lung cancer	[211,212]
BTN3A3	ORF3	- Polymorphisms of this gene may alter susceptibility to HCV infection - Potent inhibitor of avian influenza A virus (but not human IAV) by restricting its replication	[197,213]
CAMP	ORF3	- Inhibits DENV-2 at the stage of entry into cells by binding E protein - Inhibits infection of recombinant virus pseudotypes with Ebola virus (EBOV) glycoprotein and wild-type EBOV by targeting the endosomal entry step - Differentially regulated after IAV infection	[214,215,216]
CD6	ORF3	- Overexpressed in CD8+ T-cells in lymphoid tissues and BAL from chronic SIV infection	[217]
CUL2	ORF3	- NLRC5 recruits CUL2 to ubiquitinate NS3 protease and degrade it, thereby restricting DENV infection - CUL2 knockdown inhibited SAMHD1 loss during CMV infection, which functions as a restriction factor - Vif protein of Bovine immunodeficiency virus (BIV) interacts with CUL2 to degrade restrictive bovine A3 (APOBEC3) proteins	[218,219,220]
AGO2	ORF3	- Upon SARS-CoV-2 infection, associates with viral miRNA-like small RNA, CoV2-miR-O7a, and may repress mRNAs, such as BATF2, to evade the IFN response - Inhibition of AGO2 results in decreases encephalitic alphavirus replication - AGO2 knockout promotes Seneca Virus A replication - H5N1 influenza viral infection reduces AGO2 distribution in A549 cells, to promote IFN-β expression and increase host survival	[221,222,223,224]
FXYD2	ORF3	- Upregulated in HIV-2-infected cells, downregulated in HIV-1-infected PBMCs	[225]
H3C8	ORF3	- Histones mobilized during HSV-1 infection and relates histone dynamics to assembly of viral chromatin - VZV IE63 protein interacts with ASF1 and increases its binding to H3C8, which may help regulating transcription of viral or cellular genes during infection	[226,227]
H4C1	ORF3	- Binds to IAV and aggregates viral particles; potentiates IAV induced neutrophil respiratory burst responses - TIP60 inhibits HBV replication and localized HBV cccDNA chromatin complex catalyzed H4C1 acetylation. This all might regulate HBV chromatin structure	[228,229]
IL1R2	ORF3	- May play a crucial role in innate and adaptive immune responses in pathogenic avian influenza virus - High expression positively correlated with viral shedding time in SARS-CoV-2 patients	[230,231]
KIF18A	ORF3	- Expression of KIF18A is increased in host cells after IAV infection, which provide a beneficial role in replication and a potential therapeutic target	[232]
KIR3DL2	ORF3	- Could be associated with immunity against HBV infection - Expression may be associated with more rapid progression of chronic HCV fibrosis in patients	[233,234]
LAIR1	ORF3	- Chronic EBV infection is associated with low LAIR expression on NK cells - LAIR knockout mice show enhanced airway inflammation and increased neutrophil and lymphocyte recruitment after RSV infection	[235,236]
MMP2	ORF3	- Together with MMP9, it mediates sCD100 release, which has an important role in regulating intrahepatic anti-HBV CD8 T-cell responses - Increased in long-term experimental HSV encephalitis	[237]
NAA60	ORF3	- N-terminal acetyltransferase has proviral properties during IAV infection by interfering with interferon alpha signaling	[238]
NPAS4	ORF3	- Expression level of this transcription factor severely decreased after ZIKV infection of fetal murine neurons	[239]
PIWIL4	ORF3	- Promotes HIV-1 latency by imposing repressive marks at the HIV-1 5′ long terminal repeat	[240]
RAB43	ORF3	- Important for HSV-1 assembly	[241]
RAB9A	ORF3	- Differentially expressed in lymphoblastoid cell lines after EBV infection compared to resting B cells - Mediator of HPV16 trans-golgi network transit during infection - Required for replication of HIV-1, filoviruses and measles virus, probably associated with late-endosome-to plasma membrane vesicular transpart in viral assembly	[242,243,244]
SEMA4D	ORF3	- miR-125b (which is downregulated in J avian leukosis virus) and its target SEMA4D play an important role in aggressive growth of HP45 cells induced by avian leukosis viruses - loss of SEMA4D expression might play an important role in dysfunctional immunity in HIV-1 infection - IFN-alpha enhanced SEMA4D/Plexin-B1/B2 interactions plays an important role in promoting NK functions in chronic hepatitis C patients	[245,246,247]
STAU2	ORF3	- STAU2 interacts with NS1 from avian influenza virus to promote viral replication by enhancing NS1 mRNA nuclear export	[248]
TREX1	ORF3	- 3′-to 5′ DNA exonuclease, digests excess HIV DNA to inhibit IFN expression	[249]
MSI2	ORF4	- Promotes translation of multiple IRES-containing oncogenes as well as HCV IRES; inhibition of MSI2-RNA binding reduced HCV IRES activity, viral replication and liver hyperplasia in humanized mice	[250]
CAMK2A	ORF4	- HBV replication suppresses CAMK2 activity, and CAMK2 upregulation suppresses HBV replication from cccDNA via AMPK and the AKT/mTOR pathway - EV71 VP1 protein activates CAMK2A to phosphorylate vimentin, which is required for virus replication - African swine fever virus results in activation of CAMK2A and phosphorylation of vimentin, which may be necessary for cage formation surrounding virus factories	[251,252,253]
DDAH2	ORF4	- DDAH2 induces nitric oxide and Drp1 activation, which blocks innate immune responses mediated by MAVS upon viral infection - Differentially expressed protein after North-American type Porcine reproductive and respiratory syndrome virus (PRRSV) infection	[254,255]
LBH	ORF4	- LMP1 from EBV plays a fundamental role in NPC; LBH is downregulated in NPC tissues. LBH normally induces NPC cell cycle arrest and growth of tumors by downregulating NFkB transcriptional activity	[256]
TRAF1	ORF4	- Interacts with HEV RdRp - Lost from HIV- or LCMV-specific CD8 T cells in chronic phase of infection - TRAF1 expression may be upregulated by EBV LMP1 protein in NPC	[32,257,258]
DYNLT3	ORF4	- Interacts with L2 protein of HPV and is most likely required for transport purposes towards nucleus	[259]
GATAD2A	ORF4	- Circular GATAD2A promotes replication of H1N1 IAV by inhibiting autophagy	[260]
PPP1CC	ORF4	- Gene 7 of transmissible gastroenteritis virus (TGEV) mediates binding to PPP1CC and may counteract host antiviral response	[261]
CCT5	ORF4	- Cap protein of porcine circovirus type 2 (PCV2) could directly degrade CCT5 - Interacts with avian influenza nucleoprotein (NP). Expression elevated upon infection, promotes nuclear export of NP and viral polymerase activity	[262,263]
ZFYVE1	ORF4	- Binds SARS-CoV-2 NSP6, mediates the replication organelle–lipid droplet association required to sustain viral replication - Required for HCV genome replication, probably by the formation of omegasomes that are used by HCV to generate double-membrane vesicles	[264,265]
DDX20	ORF4	- Interacts with EBNA2 and EBNA3C proteins from EBV. It is suggested EBNA2 targets spliceosome complex to release SMN proteins after binding DDX20. EBNA3C binding to DDX20 stabilizes the protein to form a complex with p53 to block transcriptional activity and apoptosis - Interacts with HIV-1 Vpr protein	[266]
HEY1	ORF4	- Increased expression in two human thyroid cancer lines transfected with EBNA2 from EBV	[267]
CEP72	ORF4	- Differentially expressed in HBV-derived HCC tumors	[268]
CEP55	ORF4	- Upregulated after HTLV-1 and Bovine leukemia virus (BLV) infection	[269]
KLHL20	ORF4	- KLHL20 polyubiquitinates SERINC5, which determines its expression on the plasma membrane, where it is packaged into HIV-1 virions for viral inhibition or downregulation by Nef via lysosomal degradation	[270]
NAB2	ORF4	- Early growth response gene 1 (egr1) can repress HSV-1 early gene expression through the recruitment of co-repressor NAB2	[271]
RBFOX2	ORF4	- Identified as hub gene in HCC patients with HBV or/and HCV infection	[272]
SH3GLB2	ORF4	- SH3GLB2 regulates lung homeostasis and recovery from severe IAV infection	[273]
PSME1	ORF4	- Expression level decreased in PRRSV-infected alveolar macrophages	[274]
MSI1	ORF4	- Interacts with ZIKV genome and promotes viral replication	[275]
FBXW11	ORF4	- Vpu interacts with tetherin to direct its FBXW11-dependent proteasomal degradation, thereby alleviating the blockade to the release of infectious virions - NSs eliminates the antiviral kinase PKR by recruitment of SCF-type E3 ubiquitin ligases containing FBXW11 and beta-TRCP1 as substrate recognition subunits. This antagonism of PKR by NSs (nonstructural protein) is essential for efficient RVFV replication in mammalian cells	[276,277]
BTRC	ORF4	- EBV miR-BART10-3p targets BTRC and facilitates epithelial–mesenchymal transition of NPC - Interacts with HIV-1 Vpu and connects CD4 to the proteolytic machinery for degradation	[278,279]
RPS15A	ORF4	- Previously identified as interactor of IRES like element in gt-1 HEV - HBV-encoded X antigen upregulates RPS15A. This stimulates cell growth and tumor formation, potentially participating in HCC development	[280,281]
PRKAR1B	ORF4	- Upregulated after duck HBV infection in vitro	[282]
ADAR	ORF4	- Responsible for editing of Hepatitis delta virus (HDV) RNA to produce antigen-L, with key role in assembly of viral particles - HCV, VSV, Measles virus, and HIV-1 are all targets of ADAR - Has proviral or antiviral effects, editing-dependent or -independent - Enhances viral replication through suppression of EIF2AK2/PKR activation	[55,283]
CARD9	ORF4	- Upregulated in HEV-infected Huh-7 cells. - Recognition of viral RNA (VSV or EMCV) by RIG-I induces CARD9 and inflammasome signaling to produce IL-1β - CARD-deficient mice have impaired cytokine production upon LACV and CVB3 infection	[284,285,286]
PRKRA	ORF4	- ORF virus encoded protein OV20.0 interacts with PRKRA, blocking PACT-mediated PKR activation - Us11 protein of HSV-1 binds PACT to inhibit PKR activation - VP35 of Marburg virus interacts with PACT but is not necessarily related to PKR inhibition	[287,288,289]
DES	ORF4	- Interacts with the rabies virus matrix protein, which positively regulates virus infection - Theiler murine encephalitis virus (TMEV) virions associate with DES and vimentin. Following infection, the filament network rearranged into a shell like structure surrounding viral inclusion - HIV-1 protease cleaves DES besides myosin heavy chain, tropomyosin and an actin fraction, showing it can cause alterations in muscle cell ultrastructure that may be of relevance in infected individuals	[290,291,292]
IMPDH1	ORF4	- Knockdown suppresses human norovirus replication	[293]
ZMYM6	ORF4	- Downregulated in cells expressing HPV-derived oncoprotein E7	[294]
CHMP4C	ORF4	- Plays a role in the integrity of the recycling endosomal network, unveiled through the dependence of HSV1 on these membranes - Plays a role in HIV-1 budding - Depletion reduces flavivirus propagation	[295,296,297]

BDV = Borna disease virus, BEFV = Bovine ephemeral fever, BIV = Bovine immunodeficiency virus, BLV = Bovine leukemia virus, DENV = Dengue virus, EBOV = Ebola virus, EBV = Epstein–Barr virus, EV = Enterovirus, FAdV = Fowl adenovirus, GCRV = grass carp reovirus, HBV = Hepatitis B virus, HCC = Hepatocellular carcinoma, HCMV = Human cytomegalovirus, HCV = Hepatitis C virus, HIV = Human immunodeficiency virus, HPV = Human papillomavirus, HSV = Herpes simplex virus, HTLV = Human T-lymphotropic virus, IAV = Influenza A virus, IPNV = infectious pancreatic necrosis virus, JEV = Japanese encephalitis virus, LCMV = Lymphocytic choriomeningitis virus, NDV = Newcastle disease virus, NPC = Nasopharyngeal carcinoma, PCV = Porcine circovirus, PEDV = Porcine epidemic diarrhea virus, PRRSV = Porcine reproductive and respiratory syndrome virus, RSV = Respiratory syncytial virus, SIV = Simian immunodeficiency virus, SVCV = spring viremia of carp virus, TFV = Tiger frog virus, TGEV = Transmissible gastroenteritis virus, TMEV = Theiler murine encephalitis virus, VHSV = Viral hemorrhagic septicemia virus, VSV = Vesicular stomatitis virus, VZV = Varicella zoster virus, WNV = West Nile virus, ZIKV = Zika virus.
